# Dexamethasone-Induced MerTK^+/high^ M2c Macrophages Exhibit a Preference for Downregulated Gene Expression Profiles

**DOI:** 10.7150/jgen.108648

**Published:** 2025-03-31

**Authors:** Zhen-Tao Lee, Farrah Putri Salmanida, Hso-Chi Chaung, Ko-Tung Chang

**Affiliations:** 1 Department of Biological Science and Technology, National Pingtung University of Science and Technology, Pingtung 91201, Taiwan.; 2 Research Center for Animal Biologics, National Pingtung University of Science and Technology, Pingtung 91201, Taiwan.; 3 Department of Veterinary Medicine, National Pingtung University of Science and Technology, Pingtung 91201, Taiwan.; 4 Flow Cytometry Center, Precision Instruments Center, National Pingtung University of Science and Technology, Pingtung 91201, Taiwan.; 5 Center of Excellence for Metabolic Associated Fatty Liver Disease, National Sun Yat-Sen University, Kaohsiung, Taiwan.

**Keywords:** dexamethasone, adoptive cell transfer, allograft rejection, autoimmune disease, macrophages, M2c macrophages, transcriptome

## Abstract

In a prior study, adoptive cell transfer (ACT) of Dexamethasone (DEX)-induced M2c macrophages with positive expression of MerTK receptor mitigated acute allograft rejection, which was observed in the presence of apoptotic lymphocytes, while simultaneously reducing MHC-II and CD8^+^ T cells in the recipients. However, there has been limited exploration of the properties of adoptive M2c cells, leaving their potential for other applications unclear. In this study, we aimed to characterize the transcriptome profile of DEX-induced MerTK^+/high^ M2c macrophages. Notably, through the analysis of differentially expressed genes (DEGs), no significant pathway could be constructed from the upregulated DEGs. Only downregulated DEGs could facilitate KEGG construction, encompassing the role of DEX-induced MerTK^+/high^ M2c in immune tolerance. The expression of T-cell activation, pro- and anti-inflammatory cytokines modulation, leukocyte recruitment and adjustment of MHC-I/II-related proteins were entirely diminished. Nonetheless, association of these traits suggests the potential of MerTK^+/high^ M2c macrophages for use in ACT, particularly for autoimmune conditions such as rheumatoid arthritis, inflammatory bowel disease, type-I diabetes mellitus, and AGE/RAGE signaling pathway in diabetic complications. In summary, the preference for downregulated gene expression profiles in DEX-induced MerTK^+/high^ M2c macrophages affirms their potential for immunosuppressive adoptive cell therapy.

## Introduction

Adoptive cell transfer (ACT) involves deriving immunomodulatory cells from individuals or patients, inducing them into therapeutic cells, and reintroducing them into the patient's body. These cells function through immune tolerance mechanisms to suppress or treat diseases, including cancer. Immunomodulatory cells, such as T cells modified into chimeric antigen receptor (CAR) T cells, are commonly used in cancer therapy to target specific antigens. However, this approach has limitations, as it requires generating tumor-specific lymphocytes for each patient 1, 2. Recent studies have explored the potential use of macrophages in ACT for diseases characterized by macrophage migration or infiltration. In particular, conditions such as atherosclerosis exacerbated by diabetes mellitus have been investigated in this context 3, 4. As antigen-presenting cells (APCs), macrophages engulf foreign proteins, process them to be presented on their cell membrane in an antigenic manner. Through this process, macrophages play a crucial role in innate immunity, serving as cytokine producers and APCs, thereby contributing to the activation of adaptive immune response.

Macrophages located in various tissues undergo polarization in response to triggers in their environment, resulting in the formation of two distinct macrophage subtypes. The first subtype, known as classically activated or M1 macrophages, exhibits a wide range of pro-inflammatory characteristics including Interleukin-1 (IL-1), tumor necrosis factor (TNF), and IL-6. On the other hand, macrophage can differentiate into the second subtype, known as alternatively activated or M2 macrophages, which possess anti-inflammatory and immunoregulatory properties while expressing various molecules, such as Arginase1 (Arg1), Cluster Differentiation 163 (CD163), and CD206 5, 6. M2 macrophages can be classified into distinct subtypes, including M2a, M2b, M2c, and M2d, based on the environmental cues they receive 7. M2a macrophages are involved in allergy, profibrotic activity, anti-inflammatory responses, and wound healing. M2b macrophages contribute to Th2 activation, immune regulation, infection facilitation, and tumor progression. M2c macrophages play a role in immunosuppression, phagocytosis, tissue repair, and matrix remodeling. Lastly, M2d macrophages also known as tumor-associated macrophages (TAMs) are associated with tumor progression, angiogenesis, and apoptotic tissue clearance 8.

There are several approaches to modify macrophages for ACT, including *ex-vivo* polarization, gene editing, and engineering extracellular vesicles. The role of macrophages for ACT has advanced to clinical trials. Among the macrophage-based cell therapies listed on ClinicalTrials.gov, nine out of eleven that have progressed to phase 3 trials involve *ex vivo* polarized macrophages, suggesting their promising therapeutic potential compared to CAR macrophage therapies. These clinical trials utilized *ex vivo* polarized macrophages, which were reintroduced into patients to treat conditions such as chronic anal fissures, limb ischemia, stroke, cardiomyopathy, osteonecrosis, and arterial disease 9. This highlights the regenerative potential of macrophages in facilitating tissue repair and immunomodulation. However, dysfunctional macrophages can negatively affect their microenvironment. For instance, hereditary pulmonary alveolar proteinosis (hPAP) results from the inability of alveolar macrophages to clear GM-CSF-dependent surfactant accumulation in the alveoli. To address this impairment, Suzuki and colleagues 10 performed pulmonary macrophage transplantation (PMT) using *Csf2rβ* gene-corrected macrophages. Their findings showed the presence of GM-CSF receptor-β on alveolar macrophages two months post-treatment, effectively preventing mortality in mice.

While reactivating macrophages through gene editing can be applied in ACT, the *ex vivo* polarization of active pro-inflammatory macrophages into immunosuppressive cells also provides functionality and versatility for use in ACT. For instance, the adoptive transfer of DEX-induced M2c macrophages ameliorated acute liver allograft rejection and extended the life expectancy of rats 11. This outcome was attained by diminishing the transplanted liver's recognition under immune surveillance through the augmentation of the MGAT5 protein in CD8^+^ T cells. This enhancement, in turn, constrains the T cell receptor (TCR) from effectively engaging with the MHC peptide-loading complex, mirroring the behavior observed in MHC-II positive cells. Accordingly, employing M2c macrophages as immunomodulatory cells emerges as a compelling therapeutic strategy. This M2c category also comprises macrophages stimulated with IL-10, TGF-β, baicalin, or glucocorticoids, often resulting in enhanced production of MerTK (Mer receptor tyrosine kinase) 12-14. As a marker for M2c macrophages, MerTK is a type of receptor tyrosine kinase that belongs to the TAM receptor family (Tyro3, Axl, and Mer) 15, 16. Previous studies have shown that MerTK is crucial for the efficient clearance of apoptotic cells 17, 18. Additionally, exploiting the highly expressed MerTK^+/high^ M2c macrophages may alleviate non-alcoholic fatty liver disease (NAFLD) by suppressing proinflammatory cytokines and fibrosis in the liver by the reduction of CD4^+^ and CD8^+^ T cells in the peripheral blood^19^. Taken together, M2c macrophages are commonly considered deactivated macrophages, as their main features include decreased expression of pro-inflammatory cytokines, increased debris scavenging, regulation of T cells, and activation of a pro-healing functional program 14.

Nonetheless, while our understanding of Dexamethasone (DEX)-induced M2c macrophages as adoptive cells have primarily focused on their role in preventing allograft rejection aforementioned, their versatile capabilities of these *ex vivo*-polarized immunomodulatory macrophages suggest potential for broader applications. In this study, we aim to investigate the implications of DEX-induced M2c macrophages in various immune-related diseases. To accomplish this, we analyzed bulk RNA-seq gene expression profiles of DEX-induced MerTK^+/high^ M2c macrophages to uncover the underlying mechanisms. This analysis provides a genetic-level perspective on the interconnections between M2c macrophages and biological pathways, elucidating the advantages and possible disadvantages of these cells as a potential tools for achieving immune tolerance in ACT.

## Materials and Methods

### Animals

The animals were handled as previously described 19. In brief, we used 10 female C57BL/6 mice, aged from 19 to 27 weeks, obtained from the National Laboratory Animal Center (Taipei, Taiwan). The mice were housed in a controlled environment with a 12-hour light/dark cycle at 24°C and had access to sterilized food and water in their cages. The experimental procedures followed the guidelines of the Institutional Animal Care and Use Committee (IACUC no: NPUST-110-034) at the National Pingtung University of Science and Technology. Each experiment was conducted with two mice, and a total of five repetitions were performed, involving up to 10 mice.

### Preparation of MerTK^+/high^ M2c macrophages

Bone marrow-derived macrophages (BMDMs) were isolated and cultured as previously described 19. In brief, the bone marrow cells were collected and pooled from 19-27-week-old C57BL/6J mice by flushing their tibias and femurs with phosphate-buffered saline (PBS) containing 0.5% bovine serum albumin (Sigma-Aldrich, Steinheim, USA). Then, to establish MerTK^+/high^ M2c Macrophage, we closely followed the procedures described by Yang *et al.* (2020)*.* Mononuclear cells were first separated from the pooled bone marrow using Ficoll-Paque PLUS (manufactured by Cytiva, USA) according to the manufacture's instructions. After collection, a total of 1×10^5^ mononuclear cells were cultured in a 24-well plate, with each well containing Dulbecco's modified eagle medium (DMEM) (Corning, Manassas, VA, USA) supplemented with 10% fetal bovine serum (Hyclone, Utah, USA) and 20 ng/mL recombinant mouse of M-CSF (315-02) (PeproTech, Rocky Hill, NJ, USA) for a period of 7 days to differentiate the cells into M2 macrophages. Thereafter, the cells underwent two washes with PBS, and the medium was substituted with DMEM supplemented with 10% FBS and Dexamethasone (50 ng/mL) to induce macrophages' polarization from M2 to M2c subtype. The cells were subsequently incubated for a duration of 24 hours.

### Flow cytometry analysis of MerTK^+/high^ M2c macrophages

On day 8, either M2 or M2c macrophage cells were collected, washed twice with PBS, and then centrifuged at 300× g at 4 °C for 5 min to remove the supernatant. The identification of M2c type in BMDM was performed by staining the cells with PE-conjugated MerTK antibody following the manufacturer's guidelines. To exclude apoptotic cells, 5µl of 7-AAD (Biolegend, San Diego, CA, USA) was added to the cells, which were incubated on ice in the dark for 20 minutes. Unstained cells served as a negative control, while the single-stained cells were used for compensation controls.

### RNA extraction and reverse transcription quantitative real-time PCR

Total RNA from M2c and M2 macrophages collected on day 8 was extracted using Trizol reagent (Invitrogen, Waltham, MA, USA) and reverse transcribed with the iScript cDNA Synthesis Kit (Bio-Rad, Hercules, CA, USA). Quantitative real-time PCR was performed by using the KAPA SYBR® FAST qPCR Master Mix (2X) Kit and following the manufacturer's instruction (KAPA Biosystem, Wilmington, DE, USA). Target gene expression was normalized to *β-actin* (the housekeeping gene) as an internal control. The qPCR was conducted using the Rotor-Gene Q Real-Time PCR machine. The primer sequences (5'-3'; forward, reverse) are provided in Table 1.

### Library preparation and transcriptome profiling

The eukaryotic transcriptome analysis was previously described 19. In brief, 1 microgram of extracted and purified total RNAs from M2c and M2 macrophage cells collected on day 8 were used to follow the library construction process of TruSeq Stranded mRNA Library Prep Kit (Illumina, San Diego, CA, USA). The sequencing library was subsequently subjected to fragment size detection using the Agilent BioAnalyzer 2100 system (Agilent, Santa Clara, CA, USA) and library concentration measurement using the Real-Time PCR system. The quality-confirmed library will be sequenced on an Illumina NovaSeq 6000 sequencer with a specification of 150 bp paired-end reads generated by Genomics, BioSci & Tech Co., New Taipei City, Taiwan (https://www.genomics.com.tw/tw). Then, ribosomal RNA will be removed before aligning the rRNA-filtered reads to the reference genome for differentially expressed genes (DEGs) analysis. RNA sequencing was performed to assess gene expression levels in experimental and control samples. Statistical methods were then applied to determine the Fold Change (FC) and q-value/adjusted p-value significance tests. Differentially expressed genes (DEGs) were filtered using a predetermined threshold based on FC (Log_2_FC > 1 for upregulated genes, Log_2_FC < -1 for downregulated genes) and adjusted p-value (q < 0.05). Volcano plot graphs were generated using the customized and interactive volcano plot graph generator, VolcaNoseR 20. Over-representation analysis (ORA) was conducted separately for upregulated and downregulated DEGs using the transcriptome analysis method. The functional enrichment analysis of gene ontology (GO) and Kyoto Encyclopedia of Genes and Genomes (KEGG) terms among gene clusters were performed using the web-based enrichment analysis platform, ShinyGO 21. A customized background containing only genes with detectable expression was used for more accurate results. The whole analysis utilized a False discovery rate (FDR) cutoff of 0.05 with maximum pathway size of 500 and minimum pathway size of 50. Finally, Bar plot was conducted using GraphPad Prism 7 software (GraphPad Software, Inc. La Jolla, CA, USA). The datasets presented in this study can be found in online repositories (NCBI Bioproject PRJNA1032166: https://www.ncbi.nlm.nih.gov/bioproject/PRJNA1032166) (accessed on 26 October 2023).

### Statistical analysis

All results were presented as mean ± SD. Comparison among groups was made using unpaired t-test analysis. Statistical analysis was performed with GraphPad Prism 7 (GraphPad Software, Inc. La Jolla, CA, USA). p-values less than 0.05 were considered statistically significant.

## Results

### DEX-induced macrophages expressed M2c marker

**Figure 1:**
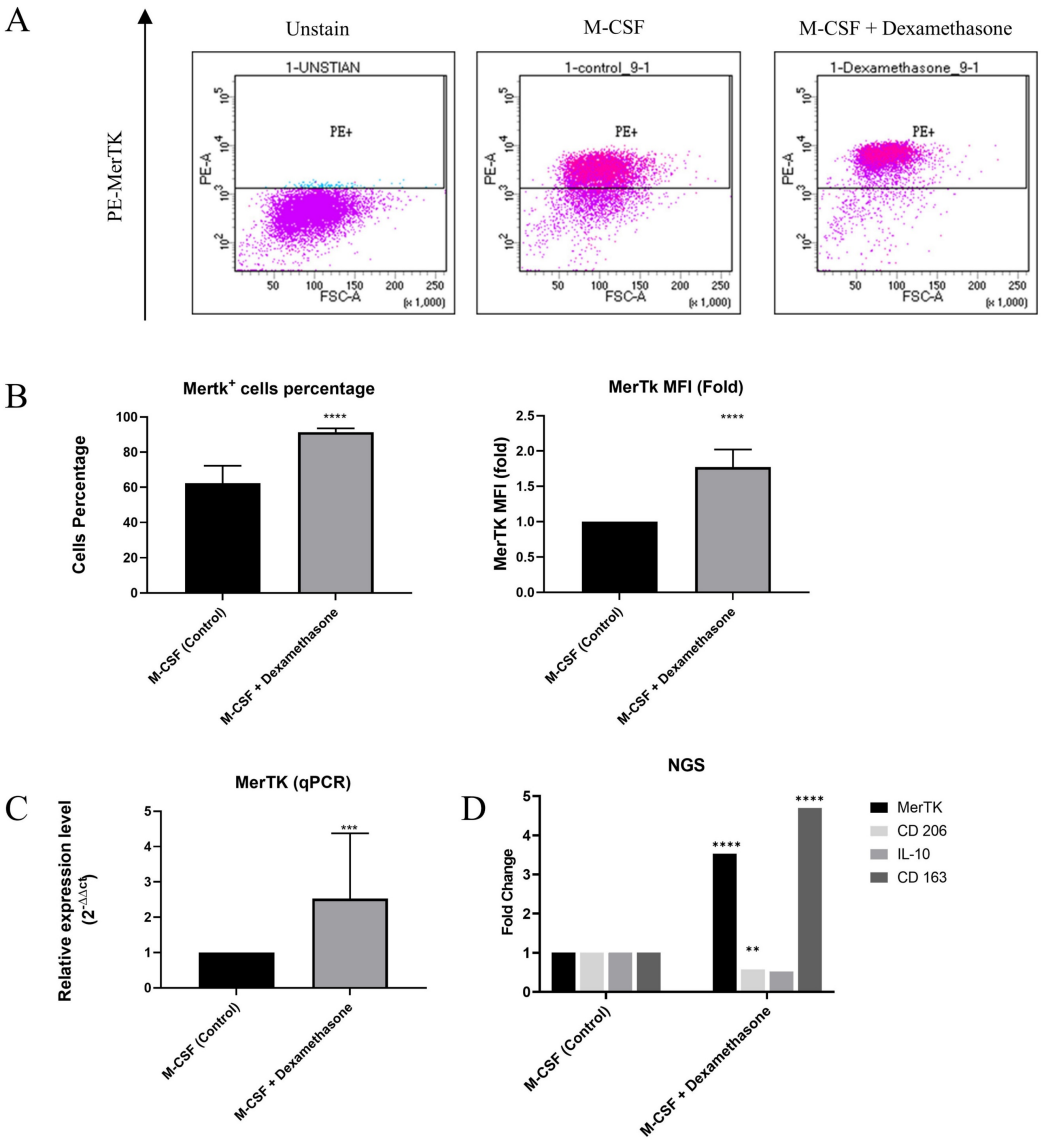
DEX-induced macrophages expressed M2c marker. (A) Surface protein MerTK is expressed on macrophages as analyzed by flow cytometry. (B) Representative data of flow cytometry analysis show the percentage of positive cells and Mean Fluorescence Intensity (MFI) of MerTK^+/high^ macrophages. (C) *MerTK* gene expression is determined by qPCR. (D) The expression of M2c polarization markers is determined by transcriptome profiling. Statistical analysis is performed by an unpaired t-test. Data are presented as the mean ± SD. (n = 10, *P < 0.05, **P < 0.01, ***P < 0.001, ****P < 0.0001).

Mononuclear cells isolated from bone marrow were differentiated into M2 macrophages by stimulation with M-CSF for 7 days. Subsequently, polarization into M2c and M2 macrophages was induced for one day with or without DEX respectively. DEX treatment significantly increased the percentage of MerTK^+^ cells and the surface protein expression of MerTK compared to M-CSF treatment alone, as indicated by mean fluorescence intensity (MFI) in flow cytometry analysis (Figure 1A and 1B). Furthermore, quantitative PCR revealed a significant upregulation of *MerTK* not only at the surface protein level but also at the gene expression level following DEX induction (Figure 1C). These experiments demonstrated that DEX induction in macrophages enhances both protein and gene expression of M2c markers, including MerTK. Following this, we employed RNA-seq to assess the expression of additional M2c markers in MerTK^+/high^ macrophages, including *MerTK*, *CD206*, *IL-10*, and *CD163*, revealing elevated expression levels compared to M2 macrophages. However, significant differences were only identified in the expression of *MerTK*, *CD206*, and *CD163* (Figure 1D).

### Transcriptome analysis of DEX-induced M2c macrophages displayed top 20 gene ontology (GO) and Kyoto Encyclopedia of Genes and Genomes (KEGG)

**Figure 2:**
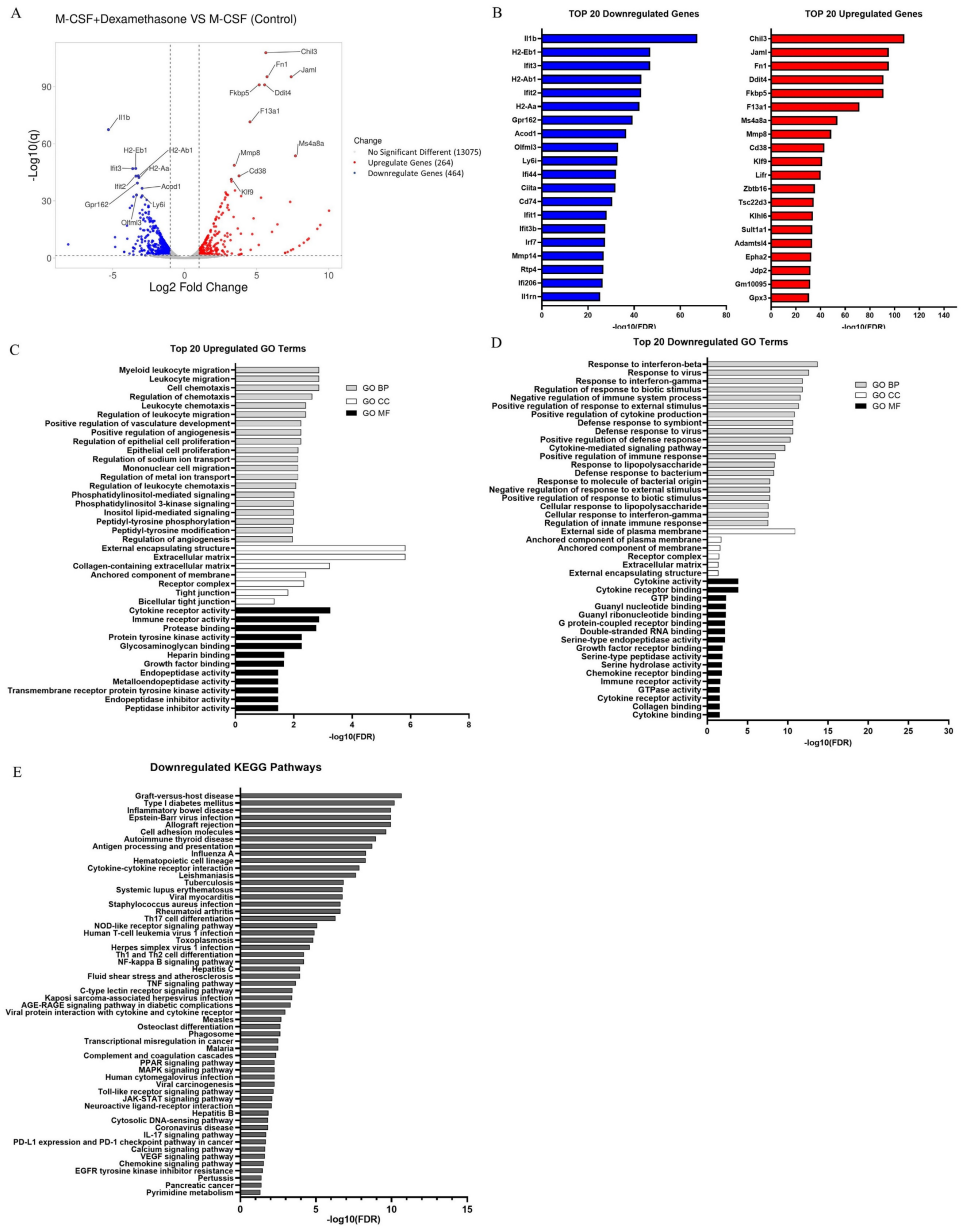
Gene Ontology (GO) and Kyoto Encyclopedia of Genes and Genomes (KEGG) pathway analysis based on the transcriptomic profiles comparison between DEX-induced M2c and M2 macrophages. (A) Volcano plot depicting the transcriptomic profiles, highlighting the top 10 DEGs. (B) Bar plots show the top 20 upregulated and downregulated DEGs. (C, D) Bar plot of the top 20 upregulated and downregulated GO term enrichments of these DEGs are shown in three functional groups: biological processes (BP), cell components (CC), and molecular functions (MF). (E) A bar plot illustrating the enrichment analysis results of 56 KEGG pathways from downregulated DEGs. The analysis is performed using ShinyGO with a false discovery rate (FDR) threshold of < 0.05 and pathway size selected between 50 to 500.

The transcriptome data from DEX-induced macrophages were compared to those of M2 macrophages induced with M-CSF alone using RNA-seq. Differential expression analysis (DEA) identified 728 genes with significant expression differences, including 264 upregulated and 464 downregulated DEGs, with only the top 10 DEGs from each group represented (Figure 2A). DEGs were filtered based on Fold Change (FC) (Log_2_FC > 1 for upregulated genes and Log_2_FC < -1 for downregulated genes) and adjusted p-values (q < 0.05) or false discovery rate (FDR). The top 20 DEGs were visualized using a bar plot (Figure 2A and 2B).

We conducted enrichment analysis on all DEGs to identify GO terms and KEGG pathways using the ShinyGO online tool. Separate analyses were performed for upregulated and downregulated DEGs. In the GO biological process category, upregulated DEGs were primarily associated with myeloid leukocyte migration (GO:0097529), leukocyte migration (GO:0097529), and cell chemotaxis (GO:0060326), whereas downregulated DEGs mainly were linked to the response to interferon-beta (GO:0035456), response to virus (GO:0009615), and response to interferon-gamma (GO:0034341). In the GO cellular component category, upregulated DEGs were mainly enriched in the external encapsulating structure (GO:0030312), extracellular matrix (GO:0031012), and collagen-containing extracellular matrix (GO:0062023), while downregulated DEGs were mainly enriched in the external side of the plasma membrane (GO:0009897), anchored component of the plasma membrane (GO:0046658), and anchored component of the membrane (GO:0031225). In the GO molecular function category, upregulated DEGs were linked to cytokine receptor activity (GO:0004896), immune receptor activity (GO:0140375), and protease binding (GO:0002020), whereas downregulated DEGs were linked to cytokine activity (GO:0005125), cytokine receptor binding (GO:0005126), and GTP binding (GO:0005525) (Figure 2C and 2D).

Unexpectedly, the KEGG enrichment analysis using the ShinyGO database revealed no significant pathways for the upregulated differentially expressed genes (DEGs) when an FDR cutoff of 0.05 was applied. However, under the same criterion, 56 pathways were significantly reduced for the downregulated DEGs. These downregulated DEGs were notably enriched in pathways related to graft-versus-host disease (mmu05332), type I diabetes mellitus (mmu04940), inflammatory bowel disease (mmu05321), Epstein-Barr virus infection (mmu05169), and allograft rejection (mmu05330) (Figure 2E).

### KEGG maps of DEX-induced M2c macrophages illustrated involvement of downregulated DEGs in regulating immune responses

**Figure 3:**
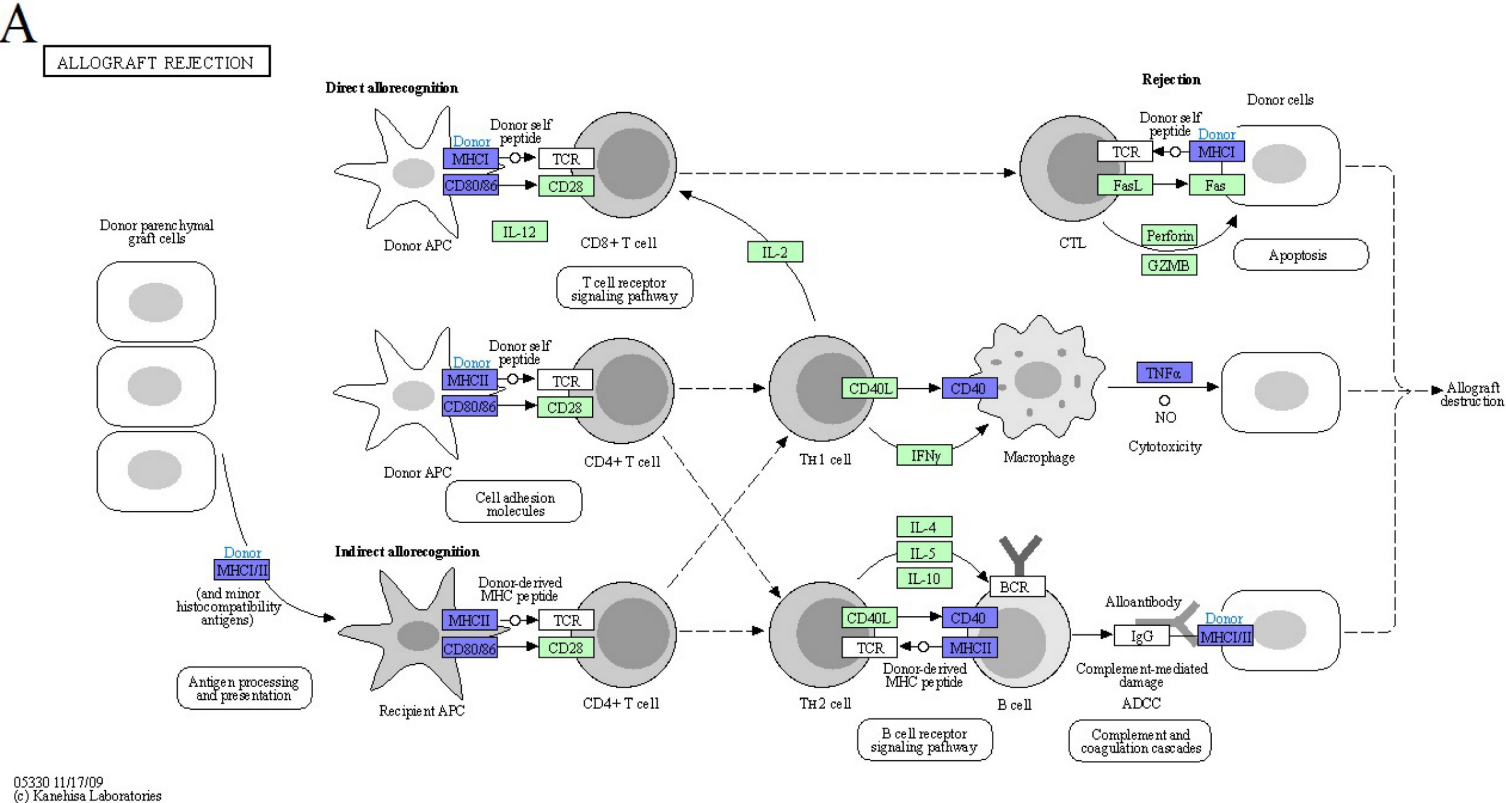

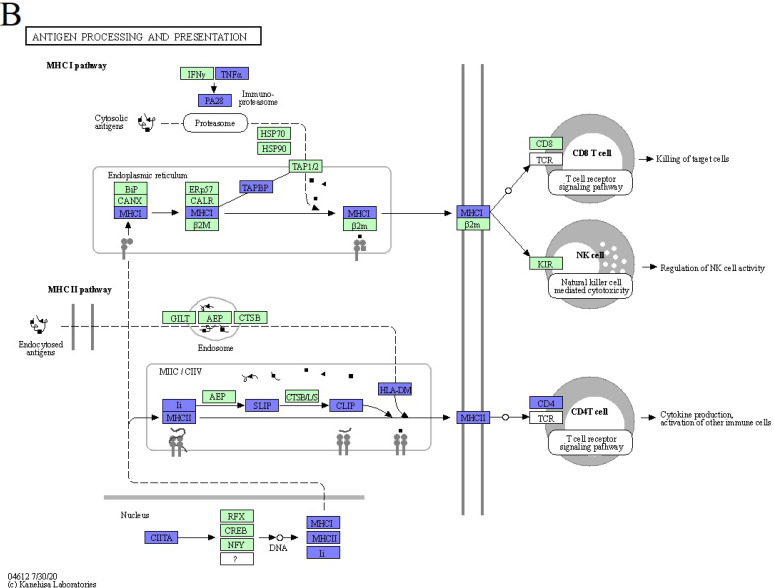

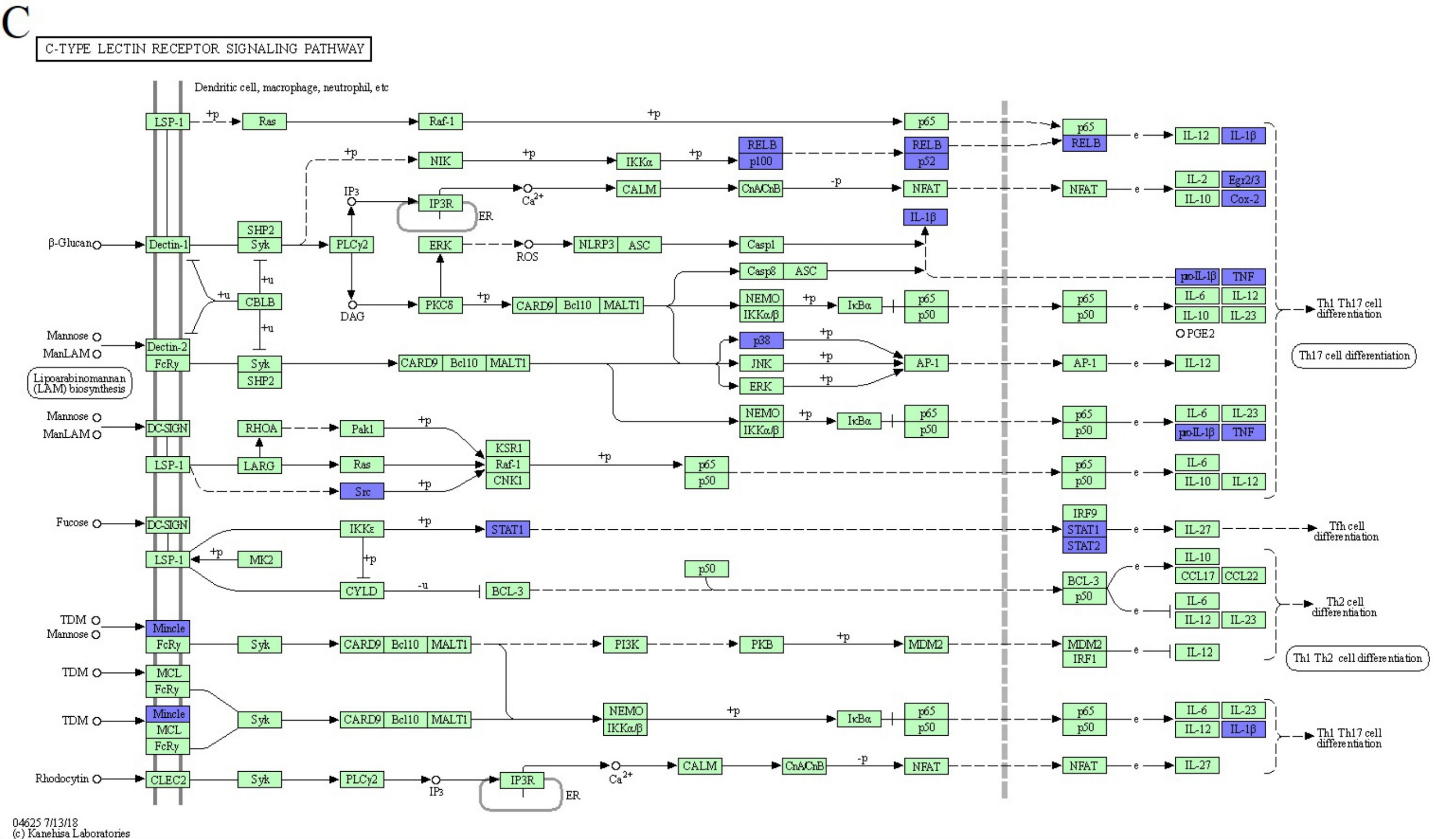

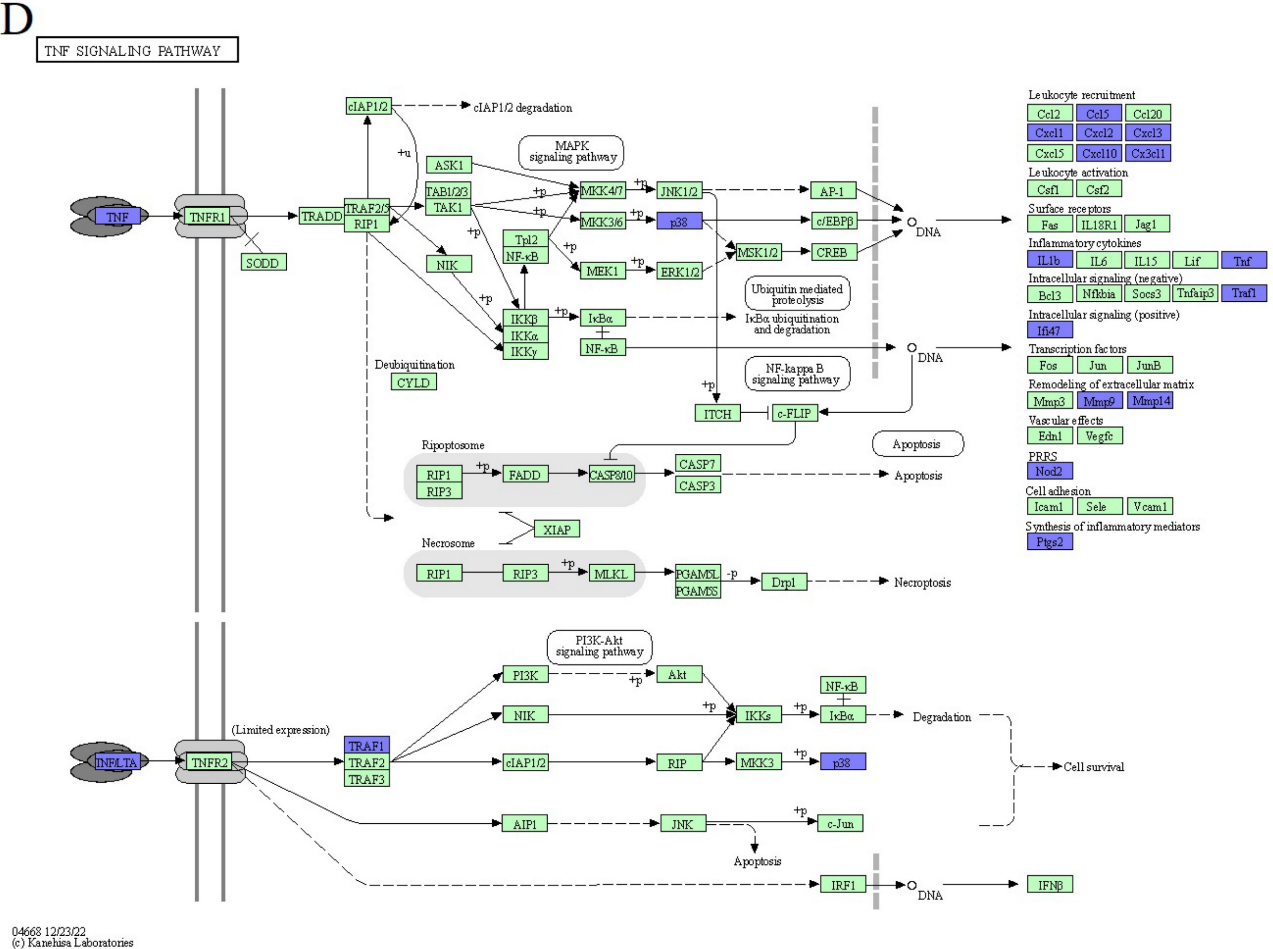

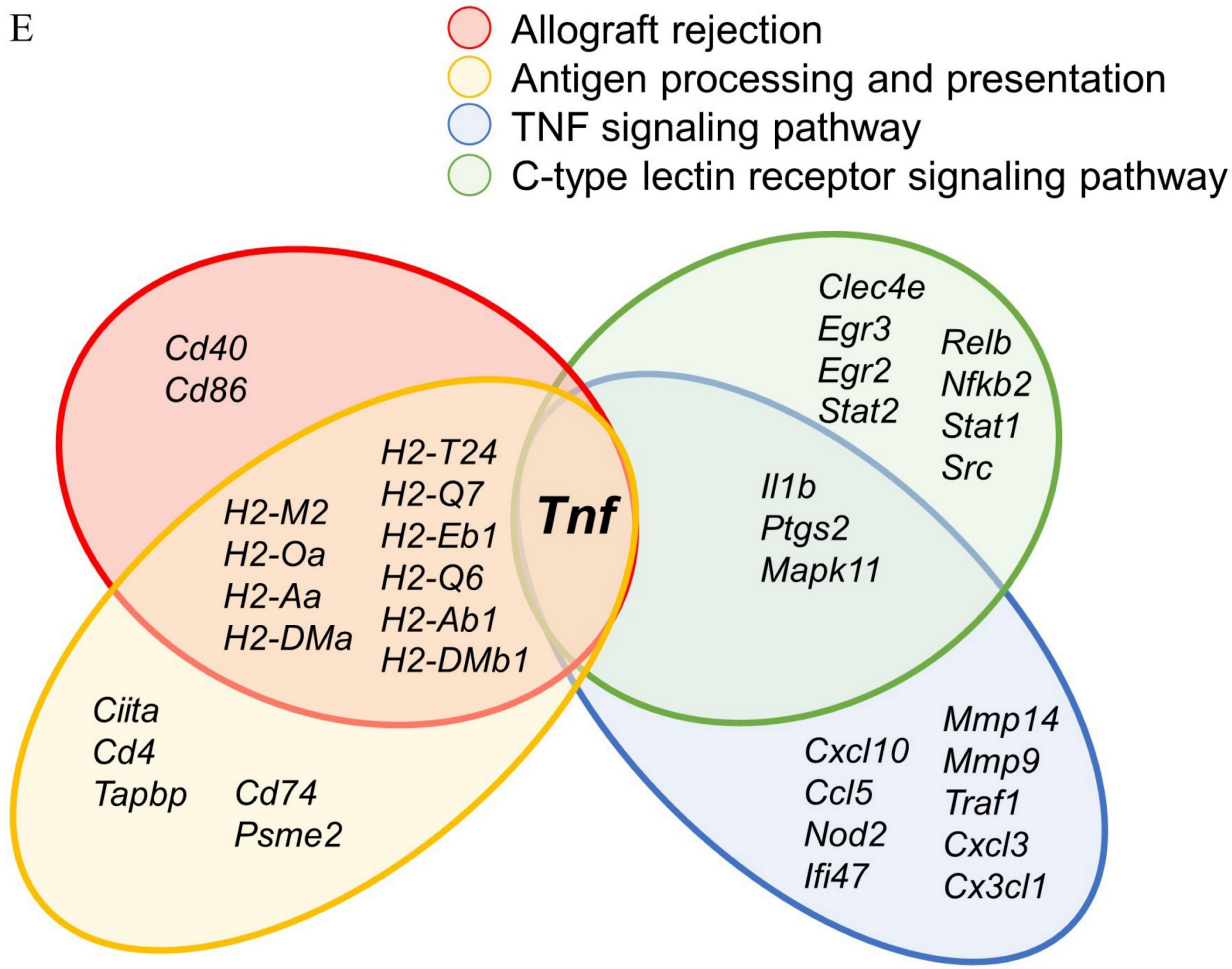
KEGG disease pathways map analysis displays downregulated DEGs influenced by DEX-induced M2c macrophages involved in anti-allograft rejection. (A) The allograft rejection pathway mediates allograft destruction. (B) The antigen processing and presentation pathways mediate T cell and NK cell activation through the major histocompatibility complex (MHC). (C) The C-type lectin receptor signaling pathway mediates helper T cells differentiation. (D) The TNF signaling pathway mediates a number of critical cell functions, including cell proliferation, survival, differentiation, inflammation, and apoptosis. The blue color indicates downregulated DEGs within the pathways. The green boxes represent the annotated protein found in KEGG database library. The white boxes are the unidentified proteins in KEGG database sequences. (E) A Venn diagram illustrates the overlap in *TNF* expression across all four signaling pathways.

We examined KEGG pathway maps derived exclusively from downregulated DEGs to identify biological pathways associated with inflammatory responses. These pathways include allograft rejection, antigen processing and presentation, the C-type lectin receptor signaling pathway, and the TNF signaling pathway. To mitigate allograft rejection after transplantation, anti-allograft rejection mechanism operates through direct or indirect means. As part of the direct method, donor macrophages polarize into the M2c subtype, attenuating host T cells activation by downregulating genes encoding MHC class I, MHC class II, and CD86. On the other hand, when employing the indirect method, polarized host M2c macrophages have the capacity to attenuate the antigen presentation of foreign antigens by diminishing MHC class II and TNF-α expression, consequently weakening inflammatory responses (Figure 3A). Within the antigen processing and presentation pathway in our M2c macrophages, downregulation of TNF-α affects immunoproteasome regulation, reducing foreign antigen cleavage and thereby lessening MHC class I presentation. Additionally, the downregulation of HLA-DR antigens-associated invariant chains (li, CLIP, SLIP) and HLA-DM impacts the assembly and subcellular trafficking of the MHC class II complex. Altered nuclear expression of the MHC class II transactivator, CIITA, further limits the transcription of MHC class I, MHC class II and li. Collectively, these results inhibit the recruitment of host NK cells, CD8^+^ T cells, and CD4^+^ T cells (Figure 3B). The C-type lectin receptor signaling pathway is the third selected pathway involved in allograft rejection, serving as a crucial pattern recognition receptor that detects pathogens and induces adaptive immune responses. Upon ligand binding, C-type lectin receptors (CLRs) activated intracellular signaling cascades, leading to the production of inflammatory cytokines and chemokines, subsequently triggering the differentiation of T helper cells. In response to signal transmission, M2c macrophages downregulate specific cytokines, including IL-1β, TNF, COX-2, EGR2, and EGR3, potentially suppressing the differentiation of Th1 and Th17 cells. Additionally, the downregulation of transcription factors like Stat1 and Stat2 influences Tfh cells differentiation (Figure 3C). Furthermore, the tumor necrosis factor (TNF) signaling pathway is activated by two receptors, TNFR1 and TNFR2. TNFR1 functions as the receptor for TNF (also called TNF-alpha), while TNFR2 interacts with both TNF and LTA. M2c macrophages downregulate TNF expression, leading to the inhibition of TNF signaling pathway products, such as CCL5, CXCL1, CXCL2, CXCL3, CXCL10, CX3CL1, IL-1β, TNF, TRAF1, IFI47, MMP9, MMP14, NOD2, and PTGS2 (Figure 3D). Notably, the convergence of these four pathways highlights the dominant role of the *TNF* gene in the anti-allograft rejection mechanism of DEX-induced M2c macrophages (Figure 3E).

### DEX-induced M2c macrophages exhibited susceptibility against parasites, bacteria and viruses

**Figure 4:**
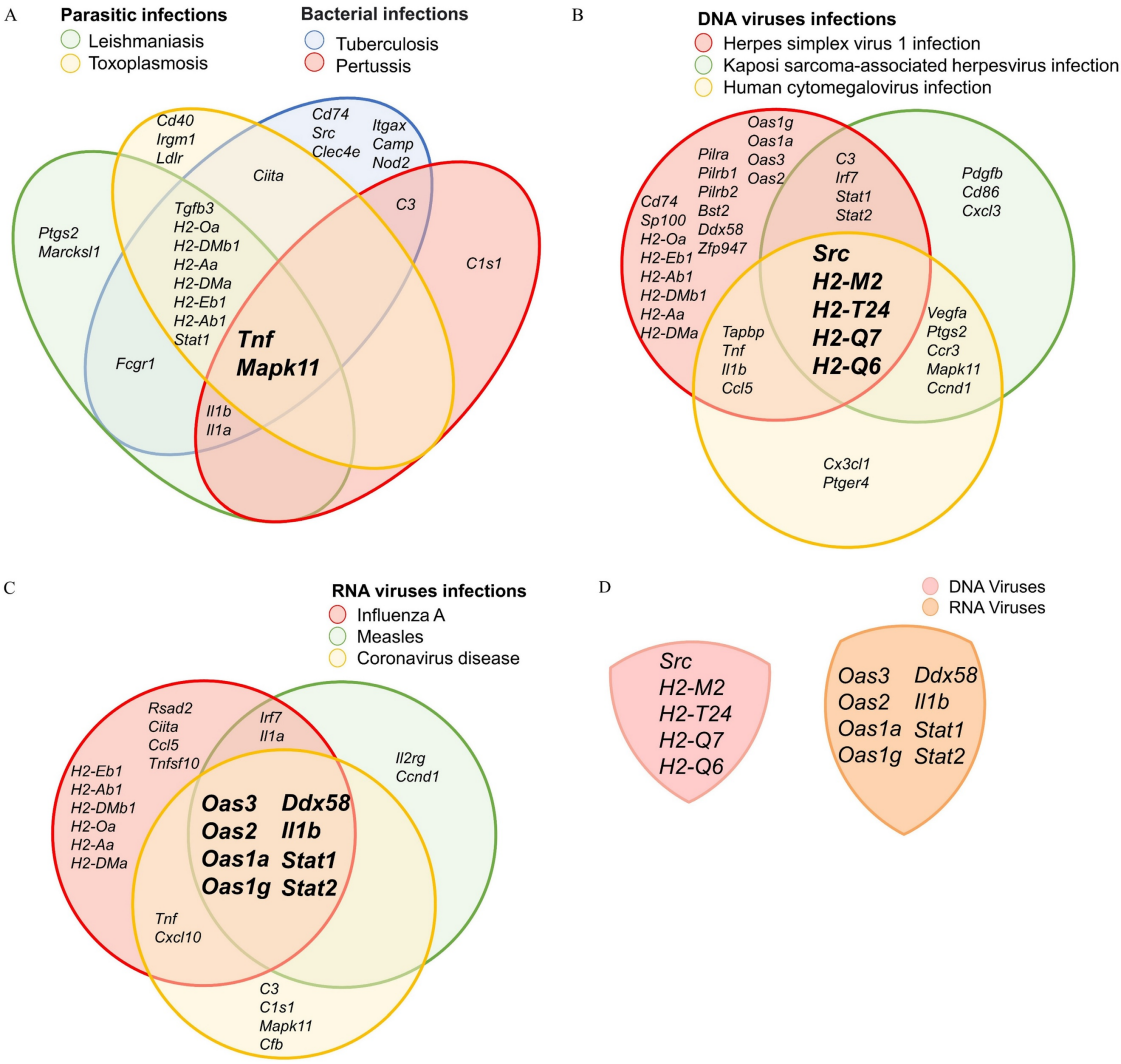
DEX-induced M2c macrophages exhibit immunosuppressive effects against parasites, bacteria, and viruses. The genes showed by overlapping the downregulated KEGG. (A) A Venn diagram generated by overlapping the four parasitic infections and bacterial infection pathways. (B) A Venn diagram generated by overlapping the three DNA virus infection pathways. (C) A Venn diagram generated by overlapping over the three RNA virus infection pathways. (D) A Venn diagram illustrates the absence of overlap between DNA and RNA viruses.

In addition to KEGG pathways associated with immune responses, we identified a link between DEX-induced M2c macrophages and an increased susceptibility to infection. This finding confirms the immunosuppressive nature of these cells, making them more vulnerable to parasitic, bacterial, and viral infections. DEGs analysis related to parasitic and bacterial infections revealed the cells' involvement in several biological pathways, including leishmaniasis, toxoplasmosis, tuberculosis, and pertussis. Analysis of overlapping downregulated DEGs in the leishmaniasis and toxoplasmosis pathways identified key association with *Tnf*, *Mapk11*, *Tbfb3*, *Stat1*, and MHC Class II-related genes (*H2-Oa*, *H2-DMb1*, *H2-Aa*, *H2-Dma*, *H2-Eb1*, and *H2-Ab1*). As a result, this could potentially contribute to an anti-inflammatory response by promoting macrophage deactivation and impairing the Th1 cell-mediated immune response (Figure 4A and Supplementary Figure S1A, S1B). Furthermore, the downregulation of *Tnf*, *Mapk11*, *C3*, *Il1a*, and *Il1b* in the tuberculosis and pertussis pathways reduces macrophages' ability to opsonize bacteria, perform phagocytosis, and initiate inflammatory responses (Figure 4A and Supplementary Figure S1C, S1D). Taken together, *TNF* and *Mapk11*, which encode TNF-α and p38β respectively, are shared between both parasitic and bacterial infection pathways. This ultimately leads to M2c macrophages losing their capacity to produce pro-inflammatory cytokines and eliminate infected cells through apoptosis (Figure 4A).

Building upon our previous analysis, we investigated the effects of DEX-derived M2c macrophages on DNA and RNA virus infections. Our findings identified three biological pathways associated with DNA virus infection, such as herpes simplex virus 1 infection, Kaposi sarcoma-associated herpesvirus infection, and human cytomegalovirus infection. These pathways highlight the enhanced ability of DNA viruses to invade host cells. This is facilitated by the inhibition of *Src* and MHC Class I genes, including *H2-M2*, *H2-T24*, *H2-Q7*, *H2-Q6* (Figure 4B and Supplementary Figure 1E, 1F, 1G). In the cases of RNA viruses, including influenza A, measles, and coronavirus disease, downregulation of genes such as *Ddx58*, *Il1b*, *Stat1*, *Stat2*, *Oas3*, *Oas2*, *Oas1a*, *Oas1g* contributes to increased susceptibility to infections. Subsequently, the OAS family encoding 2′-5′-linked oligoadenylate (2-5A) will diminish the activation of latent RNase L, thereby promoting viral replication. Simultaneously, key mediators of interferon (IFN) signaling, Stat1 and Stat2, are involved in both intrinsic antiviral defenses and adaptive immunity. Their downregulation compromises the host's ability to mount an effective response to RNA viral infections (Figure 4C and Supplementary Figure 1H, 1I, 1J). Collectively, our findings suggest that the mechanisms by which M2c macrophages promote susceptibility to DNA viral infections differ from those involved in the response of RNA viral infections (Figure 4D).

### The downregulation of *Tnf*, *Il1a*, and *Il1b* in M2c macrophages exhibited immune tolerance in autoimmune diseases

**Figure 5:**
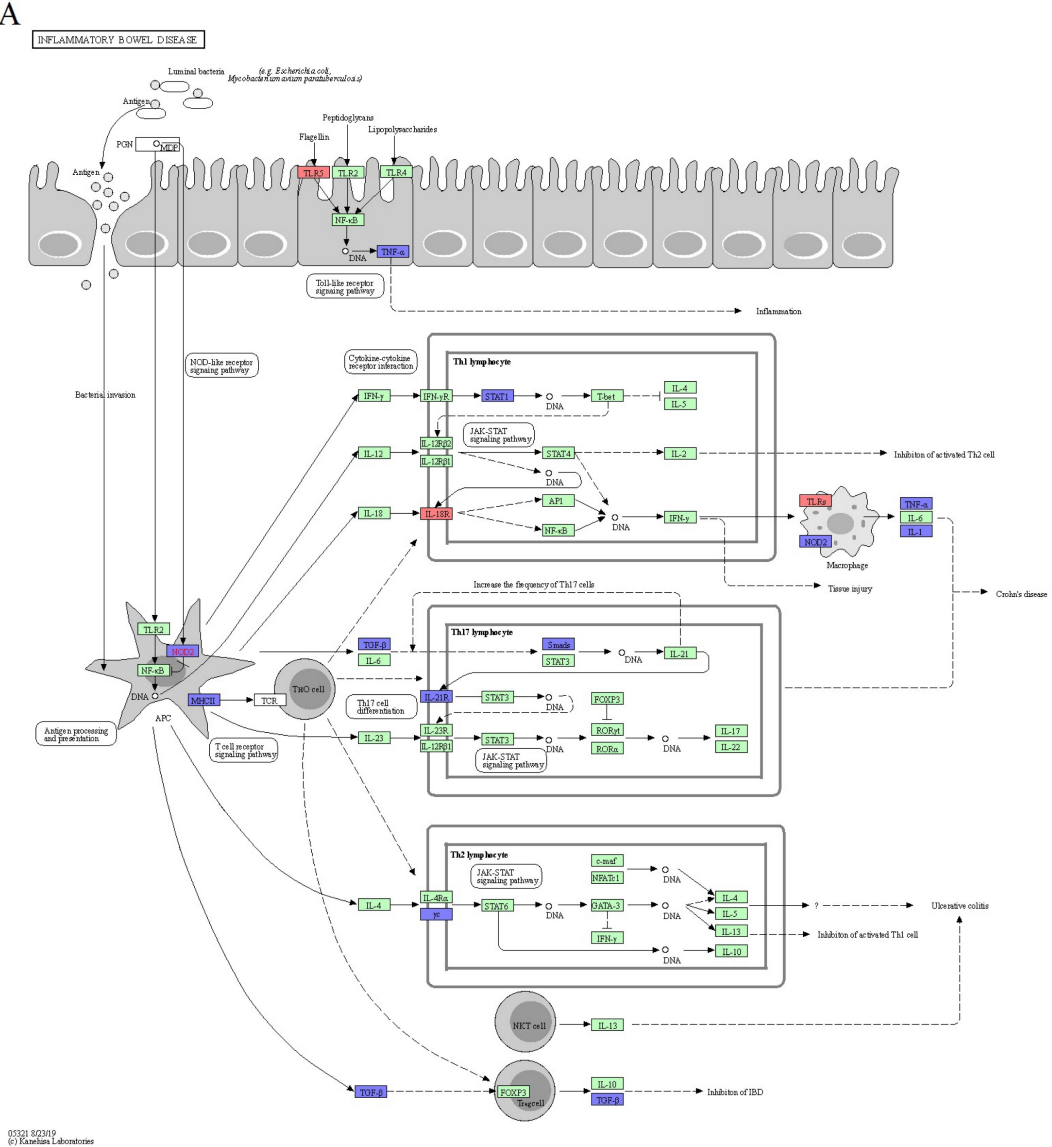

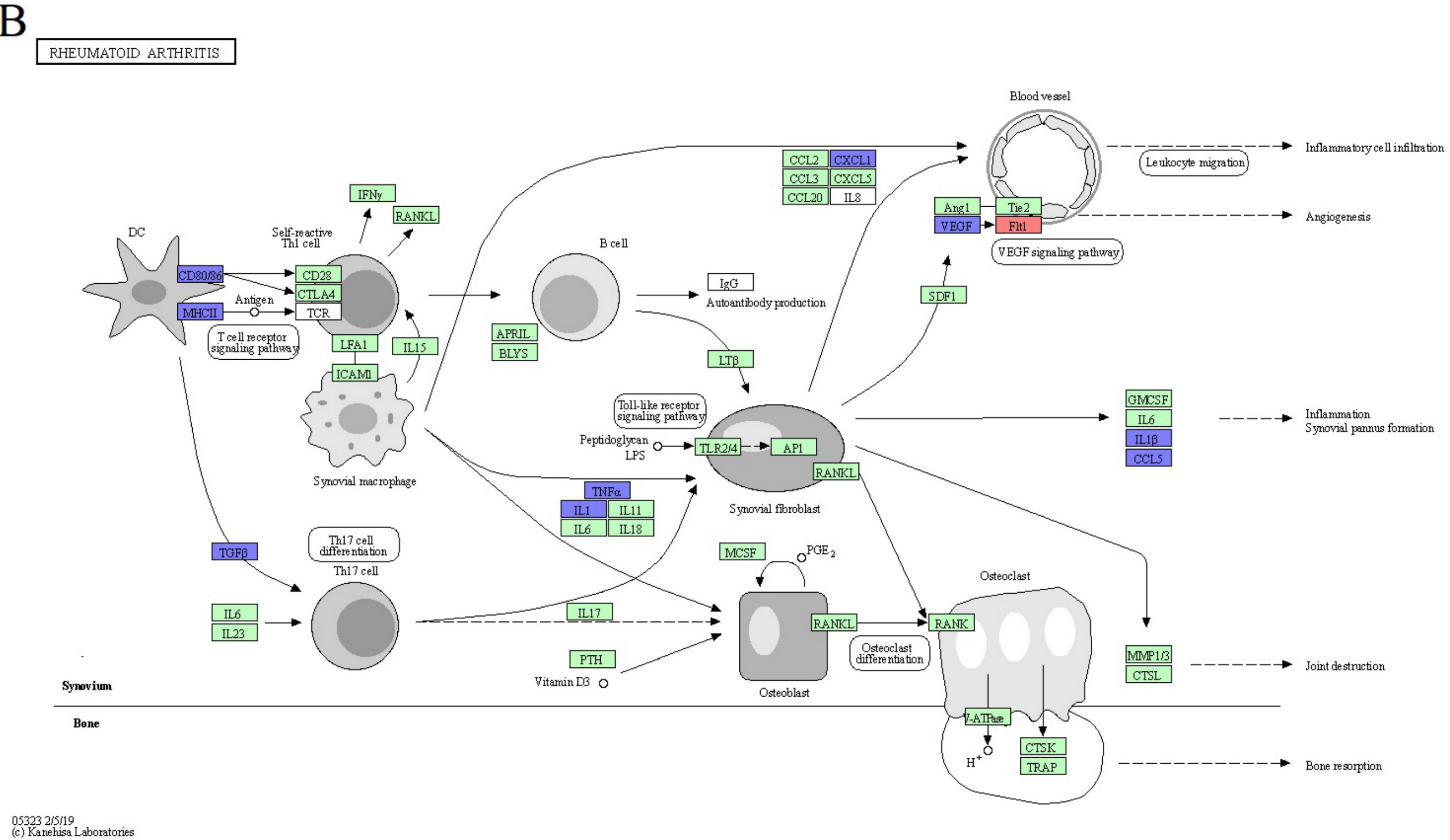

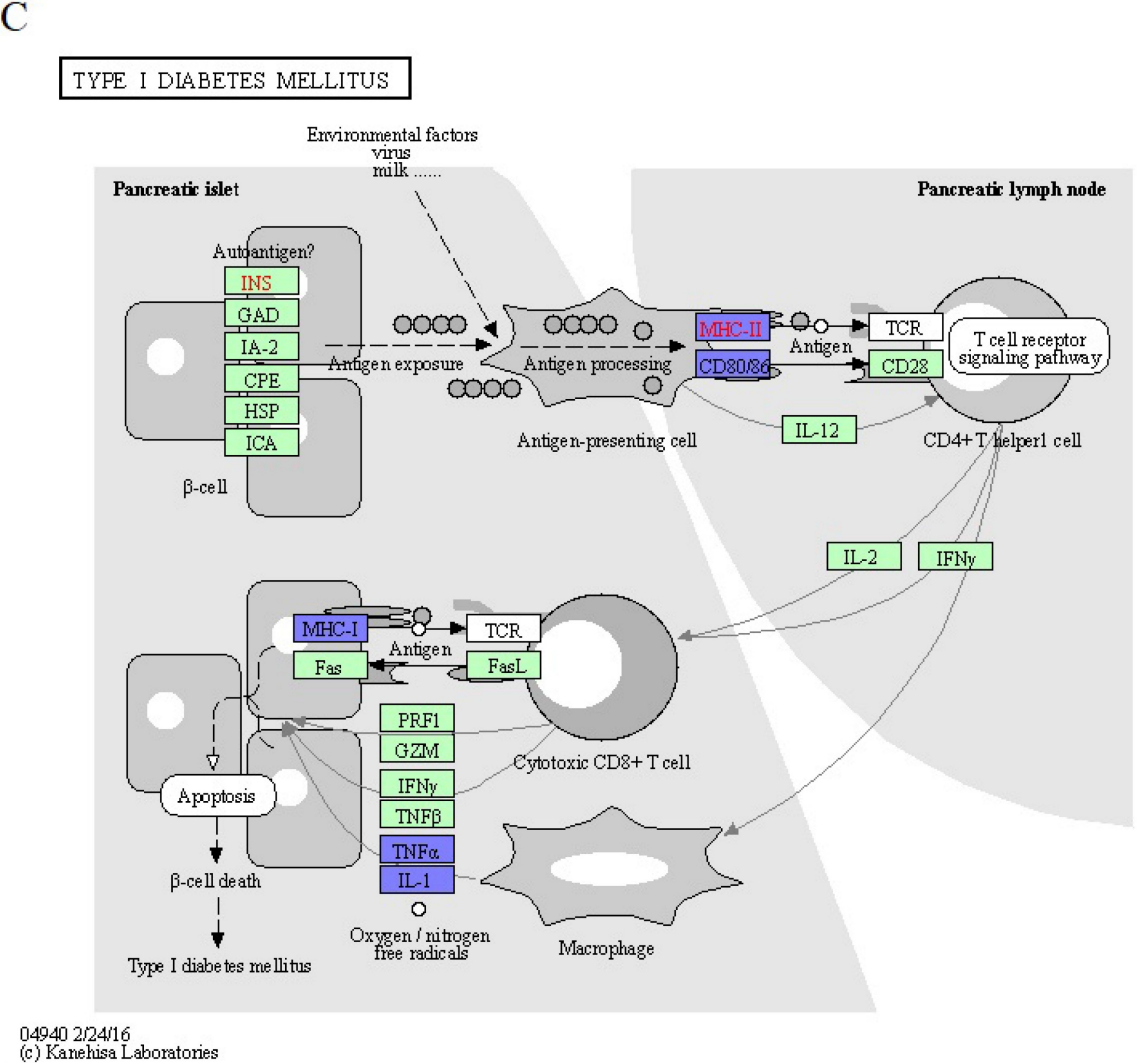

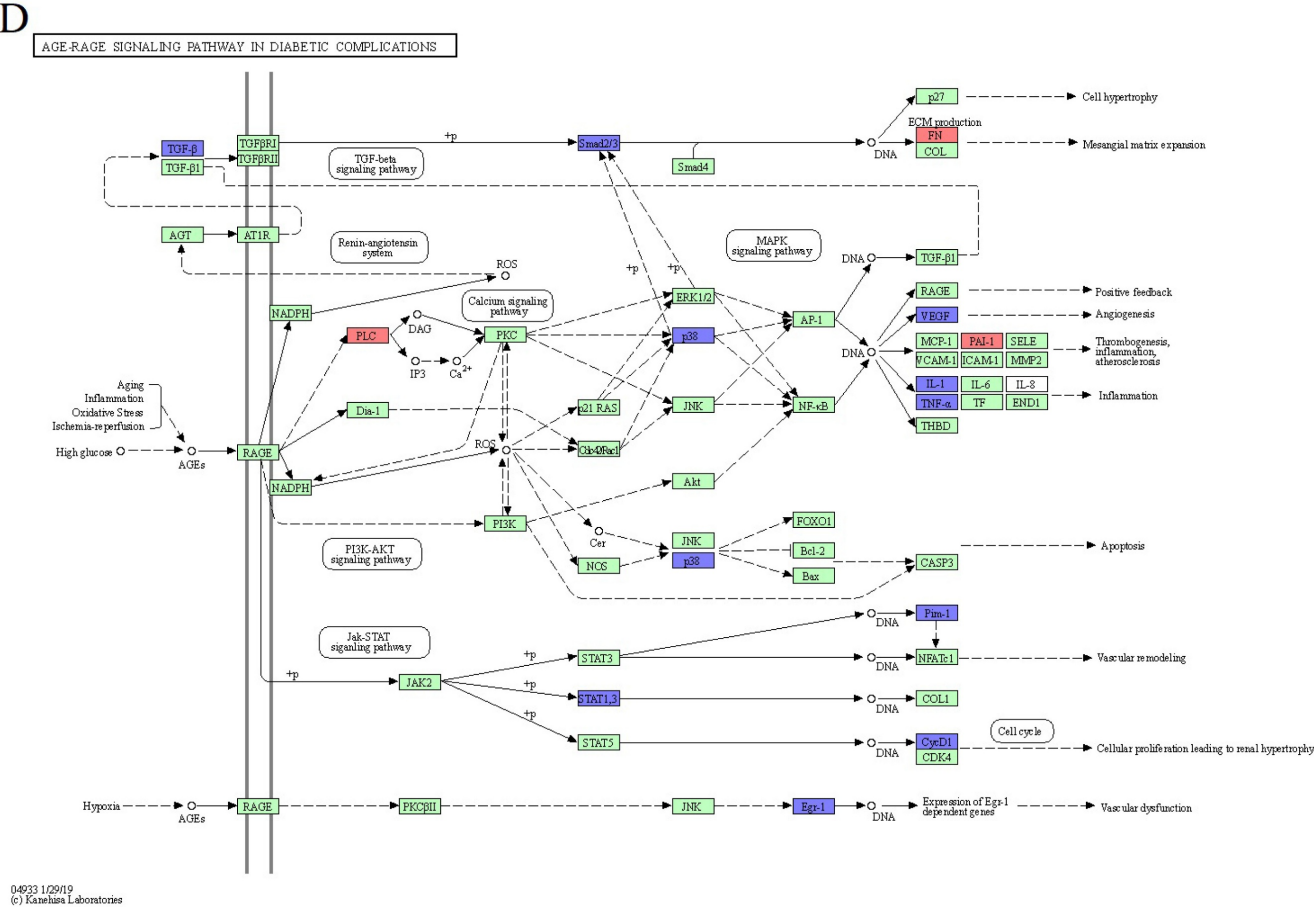

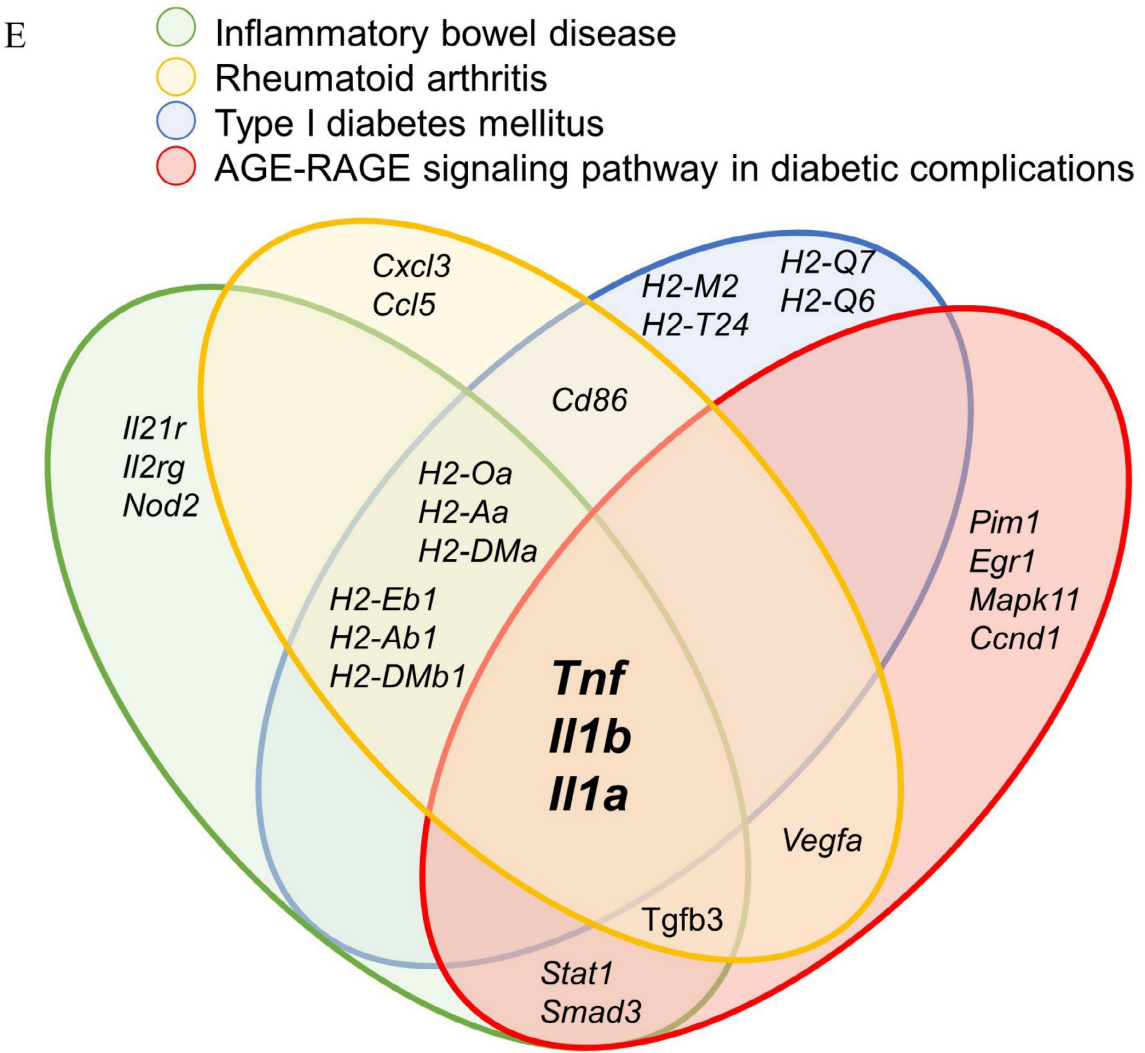
The downregulation of *Tnf*, *Il1a*, and *Il1b* in M2c macrophages exhibited immune tolerance in autoimmune diseases. The KEGG disease pathway of (A) inflammatory bowel disease, (B) rheumatoid arthritis, (C) type I diabetes mellitus, and (D) AGE-RAGE signaling pathway in diabetic complications. (E) A Venn diagram generated by overlapping the four autoimmune disease pathways highlights *Tnf*, *Il1a*, and *Il1b* expression in all. The upregulated and downregulated DEGs in the pathway are respectively denoted by red and blue colors. The green boxes represent the annotated protein found in KEGG database library. The white boxes are the unidentified proteins in KEGG database sequences.

Through an in-depth exploration of KEGG pathways, we pinpointed four autoimmune diseases that potentially modulated by DEX-induced M2c: inflammatory bowel disease, rheumatoid arthritis, type I diabetes mellitus, and the AGE-RAGE signaling pathway in diabetic complications. To determine key genes that were suppressed in these autoimmune diseases, we examined the overlap of DEGs across all pathways to reveal their relationships. This analysis revealed the downregulation of *Tnf*, *Il1a*, and *Il1b*, which played important roles in triggering autoimmune diseases. The TNF-α, IL-1α, and IL-1β proteins encoded by these genes are essential pro-inflammatory cytokines in macrophages. Inhibiting the synthesis of these pro-inflammatory cytokines can attenuate the inflammatory response associated with autoimmune disease (Figure 5A, 5B 5C, 5D and 5E). Furthermore, we identified *H2-Oa*, *H2-Aa*, *H2-DMa*, *H2-Eb1*, *H2-Ab1*, and *H2-DMb1* as critical genes encoding MHC Class II proteins. Therefore, when antigen processing and presentation cells (APCs) such as macrophages are affected, they cannot effectively activate or differentiate T helper cells, thereby influencing autoimmune disease responses.

## Discussion

A well-established approach for confirming the differentiation of cultured macrophages into M2c subtypes involves detecting specific markers such as CD163, MerTK, and IL-10 13, 18. In this study, we validated the differentiation of cultured macrophages into the M2c subtypes following DEX induction, as evidence by a significant upregulation of MerTK and CD163 (MerTK^+/high^ M2c). Despite demonstrating increased expression in the RNA-seq analysis, the *IL-10* gene did not exhibit significant differences. While previous studies have reported that IL-10 expression in M2c macrophages counteracts inflammatory responses in acute rejection 11, we hypothesized that DEX-induced M2c macrophages may achieve similar effects through alternative signaling pathways. Notably, our analysis of downregulated KEGG pathways indicates that M2c macrophages may suppress the NF-lb., TNF, and IL-17 inflammatory signaling pathways. Inhibition of these pathways corresponded with reduced production of key pro-inflammatory cytokines, including TNF-α, IL-1α, and IL-1β. These findings offer a more comprehensive understanding of M2c macrophages and their potential role in inhibiting pro-inflammatory responses through transcriptome profiling.

Under conditions of parasitic, viral, and bacterial infections, DEX-induced M2c macrophages often exhibit an increased proportion of M2b macrophages, which exacerbates susceptibility to infections by attenuating both immune and inflammatory responses 22. However, limited information is available regarding the specific response of M2c macrophages when exposed to these infections. Although our M2c macrophages were not directly infected with parasites, viruses, and bacteria, RNA-seq analysis of DEGs revealed that the most prominently expressed genes among the downregulated DEGs were associated with these infections. These genes include *Il1b*, *H2-Eb1*, *Ifit3*, *H2-Ab1*, *Ifit2*, *H2-Aa*, which respectively encode IL-1β, MHC Class II proteins, and interferon-related proteins. Correspondingly, the top three GO biological processes analysis identified a significant association between DEX-induced macrophages and these diseases, as evidenced by the downregulation of response to interferon-beta, response to virus, and response to interferon-gamma. In the context of infections, M2c macrophages exhibited downregulation of antigen presentation via MHC class I. Additionally, suppression of the OAS family, which encodes 2'-5'-linked oligoadenylate (2-5A), likely impairs latent RNase L activation. Consequently, this impediment hinders the degradation of RNA viruses, thereby promoting viral replication. Furthermore, the downregulation of Stat1 and Stat2 may disrupt the activation of interferon-stimulated gene factor 3 (ISGF3), a pivotal regulator of interferon (IFN) signaling for antiviral defense. Despite type I and type III interferons (IFNs) utilizing distinct transmembrane receptors to initiate their signaling cascades, both converge on ISGF3 as the active transcriptional regulator within the STAT signaling pathway 23, 24. Additionally, during parasitic and bacterial infections, a specific subtype of p38 mitogen-activated protein kinases, namely Mapk11 (p38β), actively participates in the production of TNF-α and COX-2 25. Inhibition of these key genes suggests that M2c macrophages may be unable to mount an effective defense against infections caused by parasites, viruses, and bacteria. Therefore, the reduced antigen presentation and suppressed pro-inflammatory cytokine production observed in M2c macrophages may render them particularly vulnerable to parasitic, viral, and bacterial infectious diseases.

Previous studies have demonstrated that M2c macrophages ameliorate acute rejection after liver transplantation, yet their broader role in autoimmune pathways remains poorly understood 11, 26. In this study, we performed RNA-seq analysis of M2c macrophages to gain deeper insights into their physiological functions and metabolic pathways in the context of autoimmune diseases. Our findings demonstrate the immunosuppressive effect of M2c macrophages, which attenuate CD4^+^ and CD8^+^ T cells activation by downregulating MHC class I and class II protein expression. Additionally, helper T cell function may be impaired due to the suppression of the C-type lectin receptor signaling pathway. As we examined the entire pathogenesis of allograft rejection, spanning from the initiation of inflammatory responses to T cells activation, we observed that downregulation of *Tnf* (TNF-α) plays as a dominant role in the anti-rejection mechanism mediated by DEX-induced M2c macrophages. Furthermore, when comparing with other autoimmune diseases, we found that the reduction of pro-inflammatory cytokines, including TNF-α, IL-1α, and IL-1β, is associated with decreases disease occurrence. Notably, the ability of M2c macrophages to downregulate MHC class II expression may also contribute to this process. Interestingly, while relevant pathways among the downregulated DEGs were identified in both GO and KEGG databases, upregulated DEGs were associated with pathways only in the GO database. Among the top four upregulated DEGs, *Chil3* is linked to immune function, whereas *Fn1*, *JamL*, and *Ddit4* are associated with cancer. *Fn1* and *JamL* serve as prognostic biomarkers for breast and thyroid cancers, lung adenocarcinoma, and gastric cancer, while *Ddit4* is a potential prognostic biomarker for colorectal cancer 27-32. These genes provide insights into the potential relationship between M2c macrophages and cancer. Unfortunately, the limitations of the study prevent further demonstration of the relationship between DEX-induced M2c macrophages and cancer. Despite these findings, our analysis of all upregulated DEGs relied solely on the KEGG database and did not identify any associated biological pathways. Therefore, we hypothesize that the upregulated DEGs may not significantly contribute to gene sets associated with specific physiological functions or metabolic pathways in the database. Our finding suggests that DEX-induced polarization of M2c macrophages does not result in the development of new characteristics, but rather leads to the loss of certain features. This loss may contribute to immune tolerance in the body.

As a synthetic glucocorticoid, DEX is widely recognized for its potent anti-inflammatory properties and extensively used in clinical settings. However, depending on dosage and duration of use, it may also be associated with certain adverse effects, including Cushing's syndrome, systemic hypertension, pathological cardiac remodelling, secondary osteoporosis, and elevated blood sugar 33-37. Therefore, our study presents an alternative approach to DEX therapy. Rather than direct administration, our findings propose an alternative *ex vivo* approach to DEX therapy, which macrophages are first polarized into the M2c subtype using DEX and then transplanted back into the body to enhance immune tolerance. This method, referred to as adoptive cell transfer (ACT), may enable a more controlled suppression of T cells and minimize the adverse effects caused by systemic DEX administration.

## Conclusion

In summary, we present a transcriptome analysis of DEX-induced M2c macrophages and confirm their functionality at the cellular level in response to DEX. According to our results, DEX-induced M2c macrophages demonstrate potential to ameliorate pro-active inflammatory diseases at the site of injury while mitigating the systemic side effects associated with systemic DEX administration. Furthermore, we affirm an *ex vivo* approach for adoptive M2c macrophages preparation that is reproducible for practical use in the future.

## Supplementary Material

Supplementary figure.

## Figures and Tables

**Table 1:** Primers used for quantitative real-time PCR.

Genes	Sequences (5'- 3')	Reference
*β-actin*	F'- AGACTTCGAGCAGGAGAT R'- ATGCCACAGGATTCCATAC	38
*MerTK*	F'- TCCTACCTCCTGTTGCGTTT R'- ATTCACACTCTCAGGCTGCT	39
