# Supplementary material for: Dexamethasone-Induced MerTK+/high M2c Macrophages Exhibit a Preference for Downregulated Gene Expression Profiles
Source: J Genomics. 2025 Mar 31;13:24–39. doi: 10.7150/jgen.108648 (PMC11980037; doi:10.7150/jgen.108648)
Supplement: Supplementary file 1 — Supplementary figure. [file jgenv13p0024s1.pdf]

Dexamethasone-Induced MerTK$^{+/high}$ M2c Macrophages Exhibit a Preference for Downregulated Gene Expression Profiles

Supplementary Material

Supplementary Figure S1 < DEX-induced M2c macrophages exhibit immunosuppressive effects against parasites, bacteria, and viruses >

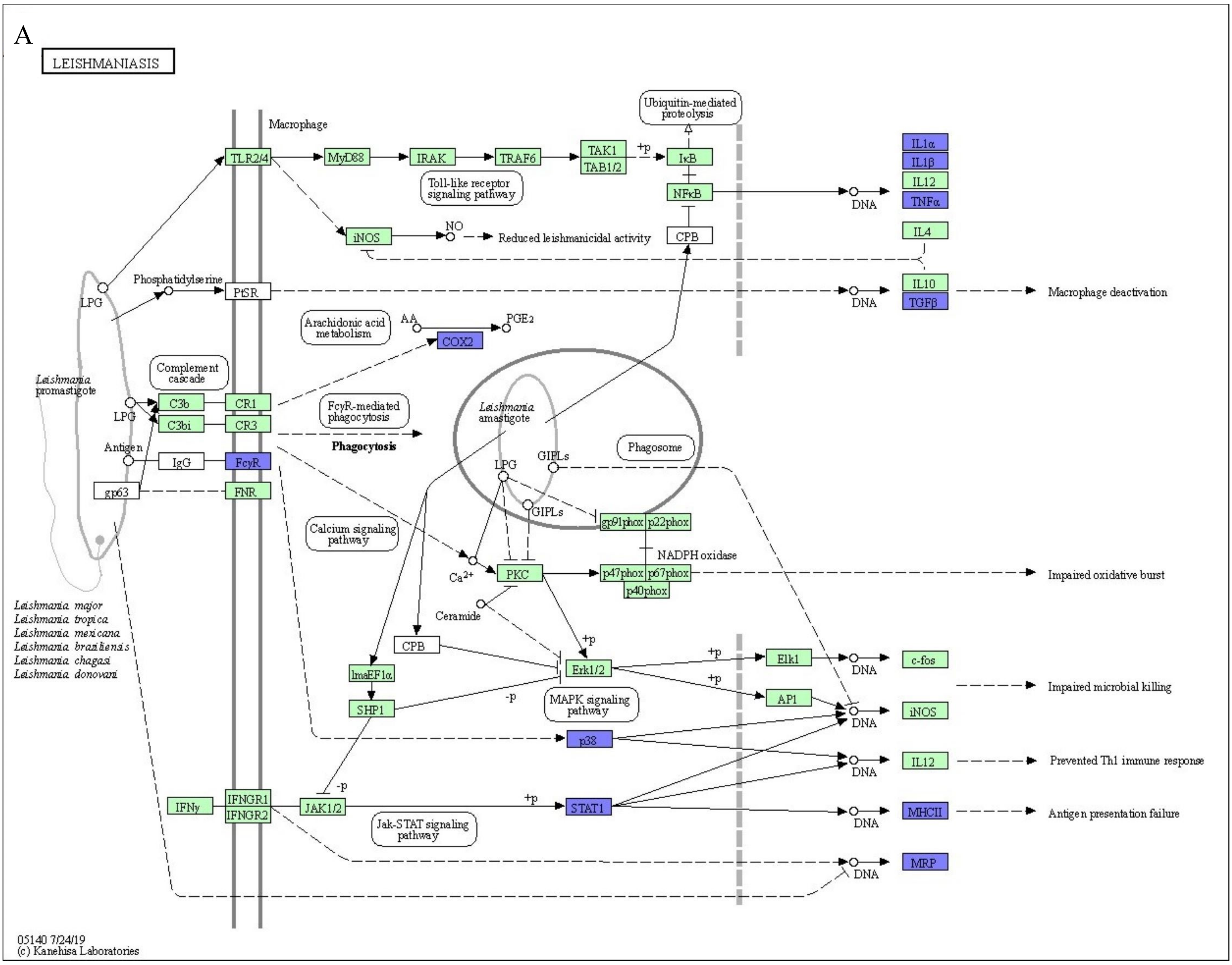

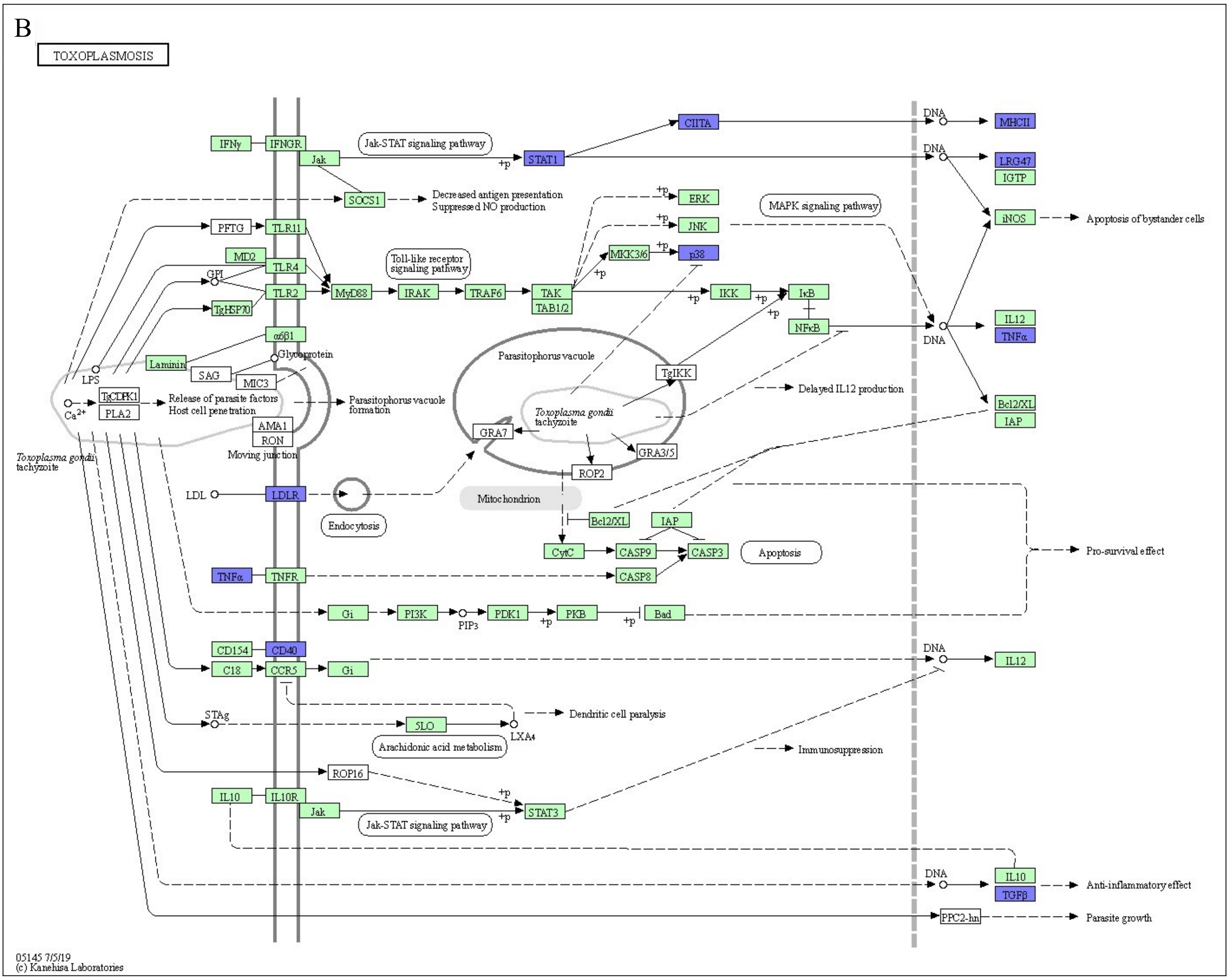

B
TOXOPLASMOSIS
IFNγ
IFNGR
Jak
Jak-STAT signaling pathway
STAT1
CIITA
DNA
MHCII
LRG47
IGTP
iNOS
Apoptosis of bystander cells
SOCS1
Decreased antigen presentation
Suppressed NO production
PFTG
TLR11
MD2
TLR4
GPI
TLR2
TgHSP70
MyD88
Toll-like receptor signaling pathway
IRAK
TRAF6
TAK
TAB1/2
ERK
JNK
MKK3/6
p38
MAPK signaling pathway
IKK
IκB
NFκB
IL12
TNFα
α6β1
Laminin
Glycoprotein
SAG
MIC3
LPS
Ca2+
TgCDPK1
PLA2
Release of parasite factors
Host cell penetration
Parasitophorus vacuole formation
AMA1
RON
Moving junction
Toxoplasma gondii tachyzoite
Parasitophorus vacuole
TgIKK
GRA7
GRA3/5
ROP2
Delayed IL12 production
Bcl2/XL
IAP
LDL
LDLR
Endocytosis
Mitochondrion
CytC
CASP9
CASP3
Apoptosis
Pro-survival effect
TNFR
CASP8
Gi
PI3K
PIP3
PDK1
PKB
Bad
+p
CD154
CD40
C18
CCR5
STAg
5LO
LXA4
Dendritic cell paralysis
Arachidonic acid metabolism
Immunosuppression
ROP16
IL10
IL10R
STAT3
TGFβ
Anti-inflammatory effect
PPC2-hn
Parasite growth
05145 7/5/19
(c) Kanehisa Laboratories

C

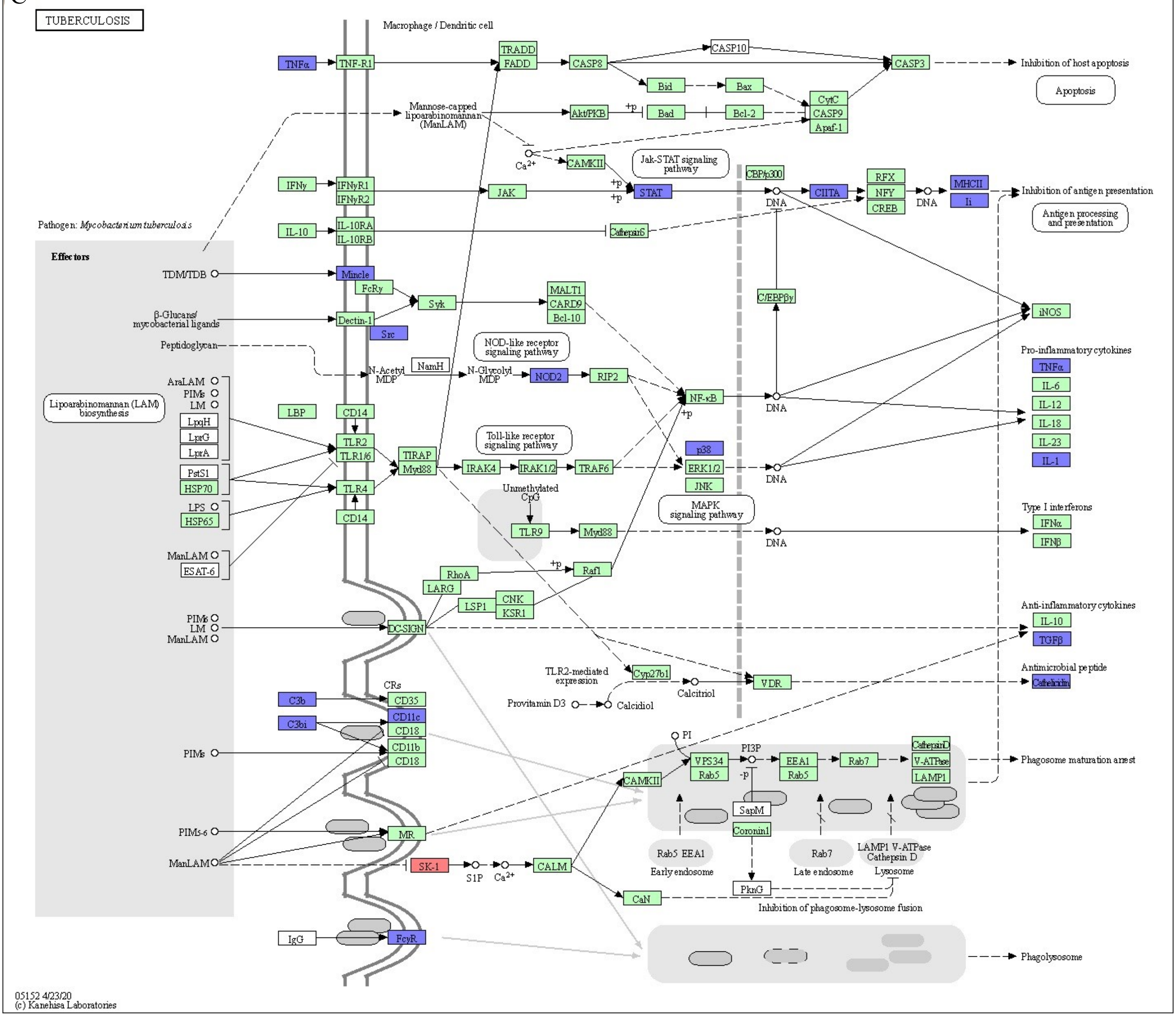
TUBERCULOSIS
Macrophage / Dendritic cell
TNFα
TNF-R1
TRADD
FADD
CASP8
CASP10
CASP3
Inhibition of host apoptosis
Apoptosis
Bid
Bax
CytC
CASP9
Apaf-1
Mannose-capped lipoarabinomannan (ManLAM)
Akt/PKB
+p
Bad
Bcl-2
Ca2+
CAMKII
Jak-STAT signaling pathway
IFNγ
IFNγR1
IFNγR2
JAK
STAT
CBP/p300
DNA
CIITA
RFX
NFY
CREB
MHCII
Ii
Inhibition of antigen presentation
Antigen processing and presentation
IL-10
IL-10RA
IL-10RB
Cathepsin S
Pathogen: Mycobacterium tuberculosis
Effectors
TDM/TDB
Mincle
FcRγ
Syk
MALT1
CARD9
Bcl-10
β-Glucans/ mycobacterial ligands
Dectin-1
Src
C/EBPβ
iNOS
Peptidoglycan
NOD-like receptor signaling pathway
NamH
N-Acetyl MDP
N-Glycolyl MDP
NOD2
RIP2
NF-κB
Pro-inflammatory cytokines
TNFα
IL-6
IL-12
IL-18
IL-23
IL-1
AraLAM
PIMs
LM
Lipoarabinomannan (LAM) biosynthesis
LpqH
LprG
LprA
LBP
CD14
TLR2
TLR1/6
TIRAP
Myd88
Toll-like receptor signaling pathway
IRAK4
IRAK1/2
TRAF6
p38
ERK1/2
JNK
PstS1
HSP70
TLR4
LPS
HSP65
Unmethylated CpG
TLR9
MAPK signaling pathway
Type I interferons
IFNα
IFNβ
ManLAM
ESAT-6
RhoA
LARG
Raf1
LSP1
CNK
KSR1
DC-SIGN
Anti-inflammatory cytokines
IL-10
TGFβ
TLR2-mediated expression
Cyp27b1
VDR
Calcitriol
Antimicrobial peptide
Cathelicidin
Provitamin D3
Calcidiol
CRs
C3b
CD35
C3bi
CD11c
CD18
CD11b
PI
PI3P
VPS34
Rab5
EEA1
Rab7
Cathepsin D
V-ATPase
LAMP1
Phagosome maturation arrest
SapM
-p
Coronin1
Rab5 EEA1
Early endosome
Late endosome
LAMP1 V-ATPase Cathepsin D
Lysosome
PIMs-6
MR
SK-1
S1P
CALM
CaN
PknG
Inhibition of phagosome-lysosome fusion
IgG
FcγR
Phagolysosome
05152 4/23/20
(c) Kanehisa Laboratories

D

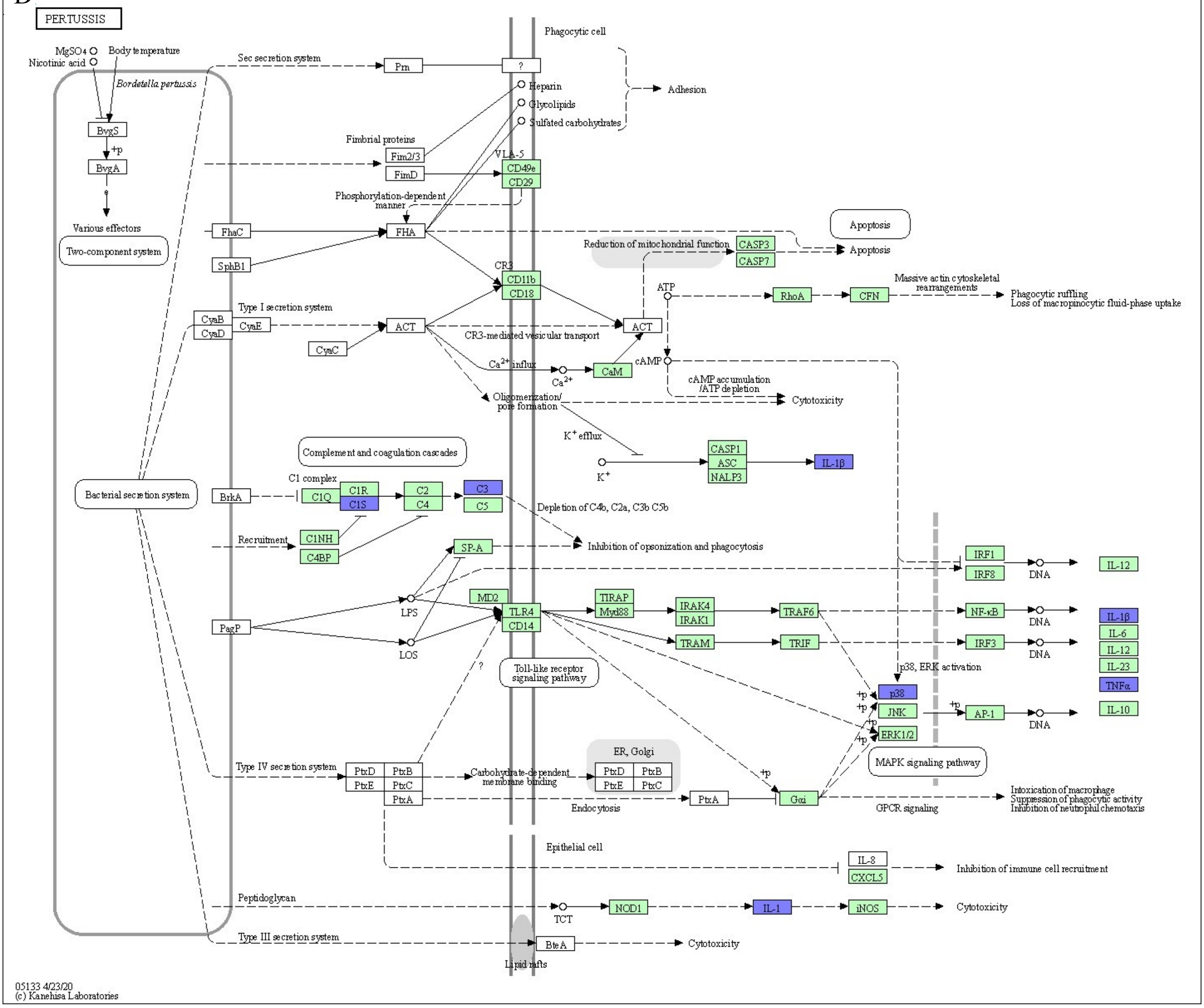

PERTUSSIS
MgSO4
Nicotinic acid
Body temperature
Bordetella pertussis
BvgS
+p
BvgA
Various effectors
Two-component system
Bacterial secretion system
Sec secretion system
Prn
?
Phagocytic cell
Heparin
Glycolipids
Sulfated carbohydrates
Adhesion
Fimbrial proteins
Fim2/3
FimD
VLA-5
CD49e
CD29
Phosphorylation-dependent manner
FhaC
FHA
SphB1
Apoptosis
Reduction of mitochondrial function
CASP3
CASP7
CR3
CD11b
CD18
ATP
RhoA
CFN
Massive actin cytoskeletal rearrangements
Phagocytic ruffling
Loss of macropinocytic fluid-phase uptake
Type I secretion system
CyaB
CyaD
CyaE
ACT
CyaC
CR3-mediated vesicular transport
Ca2+ influx
Ca2+
CaM
cAMP
cAMP accumulation /ATP depletion
Cytotoxicity
Oligomerization/ pore formation
K+ efflux
K+
CASP1
ASC
NALP3
IL-1β
Complement and coagulation cascades
C1 complex
BrkA
C1Q
C1R
C1S
C2
C4
C3
C5
Depletion of C4b, C2a, C3b C5b
Recruitment
C1NH
C4BP
SP-A
Inhibition of opsonization and phagocytosis
IRF1
IRF8
DNA
IL-12
MD2
TLR4
CD14
LPS
LOS
PagP
TIRAP
Myd88
IRAK4
IRAK1
TRAF6
NF-κB
IL-1β
IL-6
IL-12
IL-23
TNFα
IL-10
TRAM
TRIF
IRF3
Toll-like receptor signaling pathway
p38, ERK activation
p38
JNK
ERK1/2
AP-1
MAPK signaling pathway
Type IV secretion system
PtxD
PtxB
PtxE
PtxC
PtxA
Carbohydrate-dependent membrane binding
ER, Golgi
Endocytosis
Gαi
GPCR signaling
Intoxication of macrophage
Suppression of phagocytic activity
Inhibition of neutrophil chemotaxis
Epithelial cell
IL-8
CXCL5
Inhibition of immune cell recruitment
Peptidoglycan
TCT
NOD1
IL-1
iNOS
Type III secretion system
BteA
Lipid rafts
05133 4/23/20
(c) Kanehisa Laboratories

E

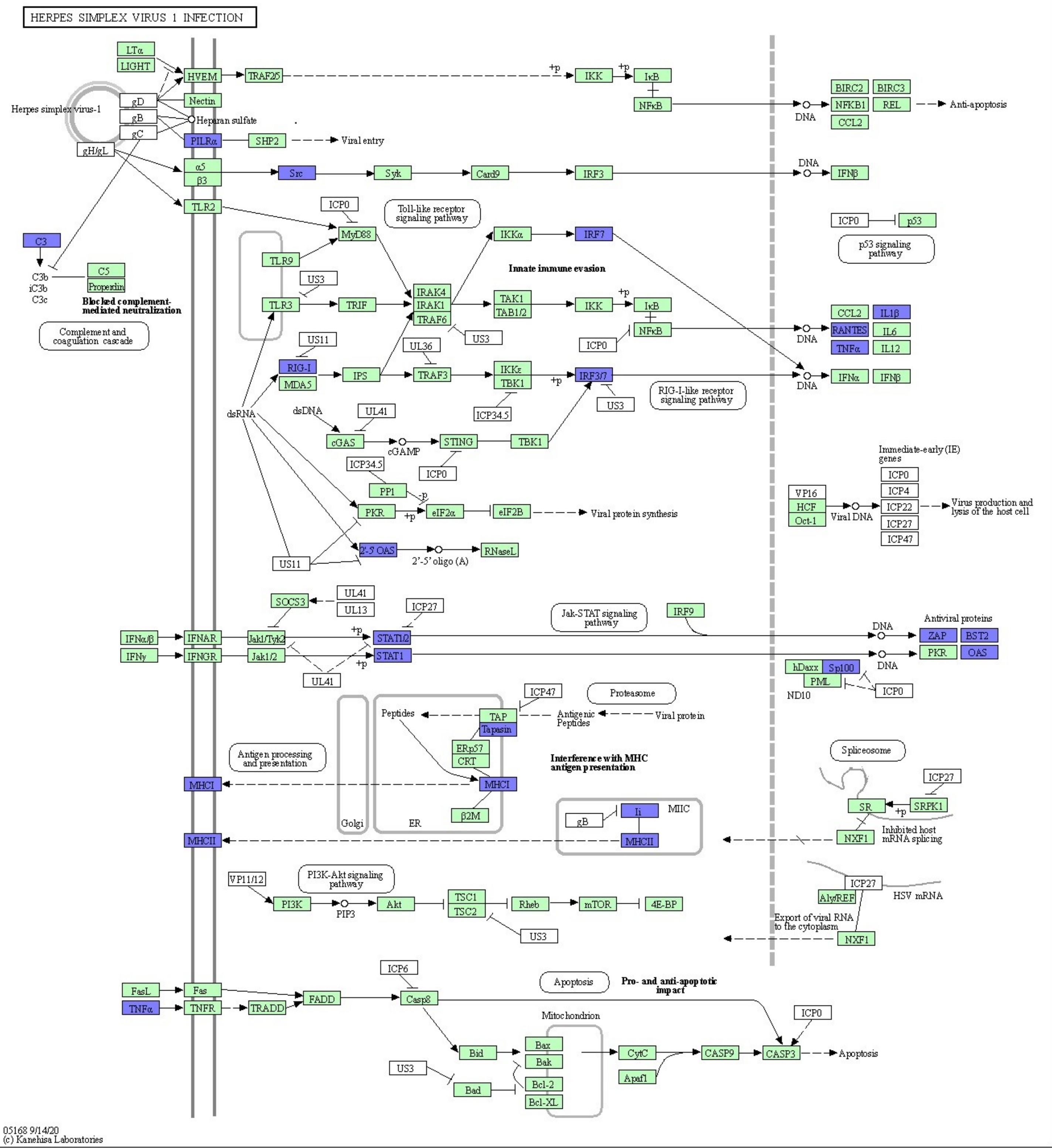
HERPES SIMPLEX VIRUS 1 INFECTION
Herpes simplex virus-1
LTα
LIGHT
HVEM
TRAF2/5
IKK
IκB
NFκB
gD
gB
gC
gH/gL
Nectin
Heparan sulfate
PILRα
SHP2
Viral entry
α5
β3
Src
Syk
Card9
IRF3
DNA
IFNβ
BIRC2
BIRC3
NFKB1
REL
CCL2
Anti-apoptosis
TLR2
ICP0
MyD88
Toll-like receptor signaling pathway
IKKα
IRF7
p53
p53 signaling pathway
C3
C3b
iC3b
C3c
C5
Properdin
Blocked complement-mediated neutralization
Complement and coagulation cascade
TLR9
TLR3
US3
TRIF
IRAK4
IRAK1
TRAF6
Innate immune evasion
TAK1
TAB1/2
CCL2
IL1β
RANTES
IL6
TNFα
IL12
US11
RIG-I
MDA5
IPS
UL36
TRAF3
IKKε
TBK1
IRF3/7
IFNα
RIG-I-like receptor signaling pathway
ICP34.5
dsRNA
dsDNA
UL41
cGAS
cGAMP
STING
Immediate-early (IE) genes
ICP4
ICP22
ICP27
ICP47
VP16
HCF
Oct-1
Viral DNA
Virus production and lysis of the host cell
PP1
PKR
eIF2α
eIF2B
Viral protein synthesis
2'-5' OAS
2'-5' oligo (A)
RNaseL
SOCS3
UL13
Jak-STAT signaling pathway
IRF9
Antiviral proteins
ZAP
BST2
OAS
IFNα/β
IFNγ
IFNAR
IFNGR
Jak1/Tyk2
Jak1/2
STAT1/2
STAT1
hDaxx
Sp100
PML
ND10
Proteasome
Peptides
TAP
Tapasin
Antigenic Peptides
Viral protein
Antigen processing and presentation
ERp57
CRT
MHCI
β2M
Golgi
ER
Interference with MHC antigen presentation
Spliceosome
SR
SRPK1
Ii
MIIC
MHCII
NXF1
Inhibited host mRNA splicing
VP11/12
PI3K-Akt signaling pathway
PI3K
PIP3
Akt
TSC1
TSC2
Rheb
mTOR
4E-BP
Aly/REF
HSV mRNA
Export of viral RNA to the cytoplasm
ICP6
Apoptosis
Pro- and anti-apoptotic impact
FasL
Fas
TNFR
TRADD
FADD
Casp8
Mitochondrion
Bid
Bax
Bak
Bad
Bcl-2
Bcl-XL
CytC
Apaf1
CASP9
CASP3
05168 9/14/20
(c) Kanehisa Laboratories

F

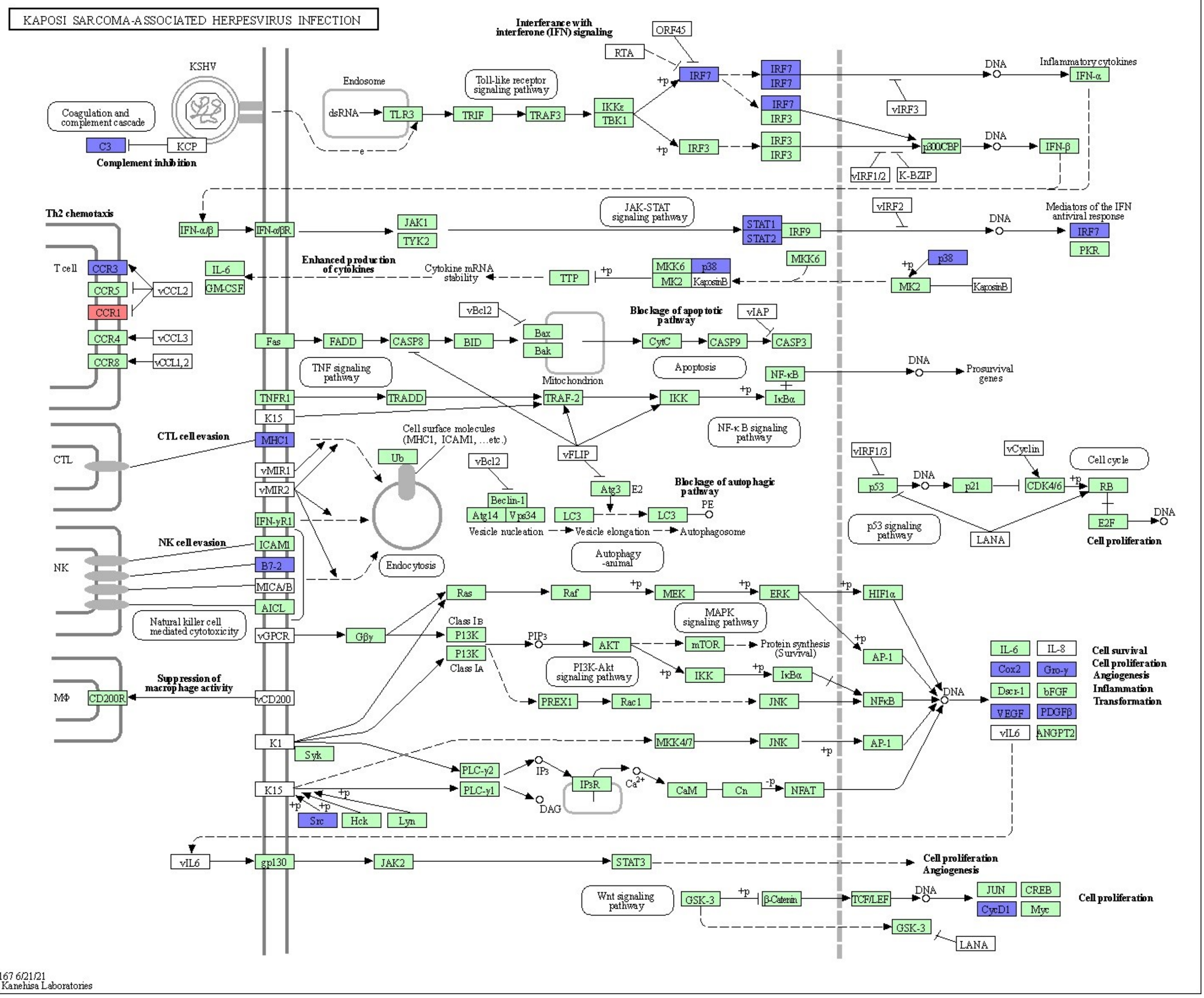
KAPOSI SARCOMA-ASSOCIATED HERPESVIRUS INFECTION
KSHV
Coagulation and complement cascade
C3
KCP
Complement inhibition
Endosome
dsRNA
TLR3
TRIF
TRAF3
Toll-like receptor signaling pathway
IKKε
TBK1
Interferance with interferone (IFN) signaling
ORF45
RTA
IRF7
IRF3
vIRF3
vIRF1/2
K-BZIP
p300/CBP
DNA
Inflammatory cytokines
IFN-α
IFN-β
Th2 chemotaxis
T cell
CCR3
CCR5
CCR1
CCR4
CCR8
vCCL2
vCCL3
vCCL1,2
IFN-α/β
IFN-α/βR
JAK1
TYK2
JAK-STAT signaling pathway
STAT1
STAT2
IRF9
vIRF2
Mediators of the IFN antiviral response
PKR
IL-6
GM-CSF
Enhanced production of cytokines
Cytokine mRNA stability
TTP
MKK6
p38
MK2
KaposinB
Fas
FADD
CASP8
BID
vBcl2
Bax
Bak
Mitochondrion
Blockage of apoptotic pathway
vIAP
CytC
CASP9
CASP3
Apoptosis
TNF signaling pathway
NF-κB
IκBα
Prosurvival genes
TNFR1
TRADD
TRAF-2
IKK
K15
NF-κB signaling pathway
CTL cell evasion
CTL
MHC1
vMIR1
vMIR2
Cell surface molecules (MHC1, ICAM1, ...etc.)
Ub
vFLIP
Atg3
E2
Blockage of autophagic pathway
Beclin-1
Atg14
Vps34
LC3
PE
Vesicle nucleation
Vesicle elongation
Autophagosome
Autophagy - animal
IFN-γR1
Endocytosis
NK cell evasion
NK
ICAM1
B7-2
MICA/B
AICL
Natural killer cell mediated cytotoxicity
vIRF1/3
p53
p21
vCyclin
CDK4/6
RB
Cell cycle
E2F
LANA
p53 signaling pathway
Cell proliferation
Ras
Raf
MEK
ERK
HIF1α
MAPK signaling pathway
vGPCR
Gβγ
Class IB
PI3K
Class IA
PIP3
AKT
mTOR
Protein synthesis (Survival)
PI3K-Akt signaling pathway
AP-1
IL-8
Cox2
Gro-γ
Dscr-1
bFGF
VEGF
PDGFβ
vIL6
ANGPT2
Cell survival
Cell proliferation
Angiogenesis
Inflammation
Transformation
Suppression of macrophage activity
MΦ
CD200R
vCD200
PREX1
Rac1
JNK
NFκB
K1
Syk
MKK4/7
PLC-γ2
PLC-γ1
IP3
IP3R
DAG
Ca2+
CaM
Cn
NFAT
Src
Hck
Lyn
+p
-p
vIL6
gp130
JAK2
STAT3
Cell proliferation
Angiogenesis
Wnt signaling pathway
GSK-3
β-Catenin
TCF/LEF
JUN
CREB
CycD1
Myc
05167 6/21/21
(c) Kanehisa Laboratories

G

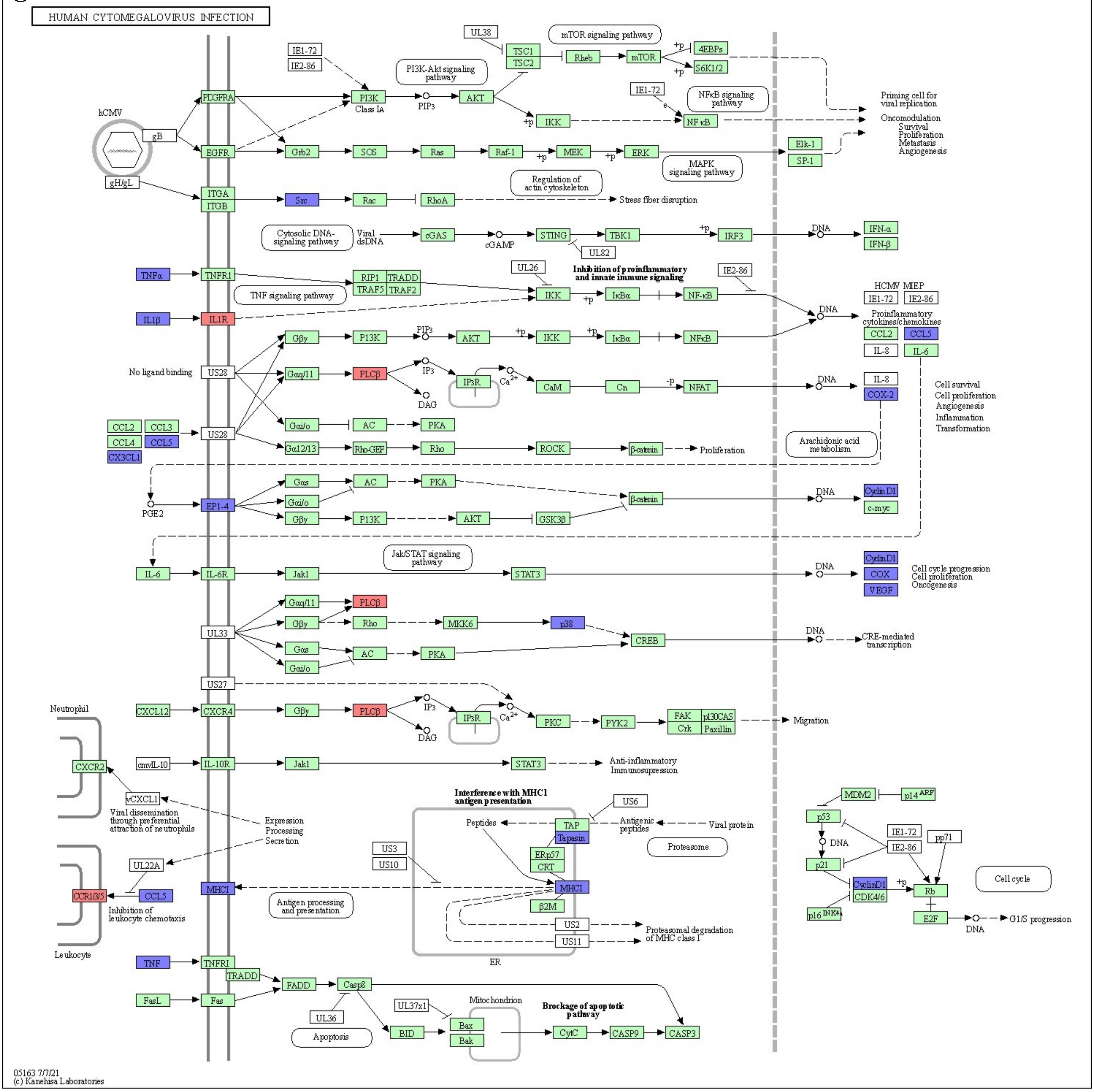
HUMAN CYTOMEGALOVIRUS INFECTION
hCMV
gB
gH/gL
PDGFRA
EGFR
ITGA
ITGB
IE1-72
IE2-86
PI3K
Class IA
PIP3
AKT
PI3K-Akt signaling pathway
UL38
TSC1
TSC2
Rheb
mTOR
mTOR signaling pathway
+p
4EBPs
S6K1/2
IKK
NFκB signaling pathway
NFκB
Grb2
SOS
Ras
Raf-1
MEK
ERK
MAPK signaling pathway
Elk-1
SP-1
Priming cell for viral replication
Oncomodulation
Survival
Proliferation
Metastasis
Angiogenesis
Src
Rac
RhoA
Regulation of actin cytoskeleton
Stress fiber disruption
Cytosolic DNA-signaling pathway
Viral dsDNA
cGAS
cGAMP
STING
UL82
TBK1
IRF3
DNA
IFN-α
IFN-β
TNFα
TNFR1
RIP1
TRADD
TRAF5
TRAF2
TNF signaling pathway
UL26
Inhibition of proinflammatory and innate immune signaling
IE2-86
IκBα
NF-κB
HCMV MIEP
IE1-72
IE2-86
Proinflammatory cytokines/chemokines
CCL2
CCL5
IL-8
IL-6
IL1β
IL1R
Gβγ
P13K
AKT
IKK
IκBα
NFκB
No ligand binding
US28
Gαq/11
PLCβ
IP3
IP3R
Ca2+
DAG
CaM
Cn
-p
NFAT
IL-8
COX-2
Cell survival
Cell proliferation
Angiogenesis
Inflammation
Transformation
CCL2
CCL3
CCL4
CCL5
CX3CL1
US28
Gαi/o
AC
PKA
Gα12/13
Rho-GEF
Rho
ROCK
β-catenin
Proliferation
Arachidonic acid metabolism
PGE2
EP1-4
Gαs
Gαi/o
Gβγ
P13K
AKT
GSK3β
β-catenin
Cyclin D1
c-myc
IL-6
IL-6R
Jak1
Jak/STAT signaling pathway
STAT3
CyclinD1
COX
VEGF
Cell cycle progression
Cell proliferation
Oncogenesis
UL33
Gαq/11
PLCβ
Gβγ
Rho
MKK6
p38
CREB
Gαs
Gαi/o
AC
PKA
CRE-mediated transcription
US27
Neutrophil
CXCL12
CXCR4
Gβγ
PLCβ
IP3
IP3R
Ca2+
DAG
PKC
PYK2
FAK
p130CAS
Crk
Paxillin
Migration
CXCR2
cmvIL-10
IL-10R
Jak1
STAT3
Anti-inflammatory
Immunosupression
vCXCL1
Viral dissemination through preferential attraction of neutrophils
Expression
Processing
Secretion
UL22A
CCR1/3/5
CCL5
Inhibition of leukocyte chemotaxis
Leukocyte
MHCI
Antigen processing and presentation
Interference with MHC1 antigen presentation
US6
Peptides
TAP
Tapasin
Antigenic peptides
Viral protein
Proteasome
US3
US10
ERp57
CRT
MHCI
β2M
US2
US11
Proteasomal degradation of MHC class I
ER
MDM2
p14ARF
p53
DNA
IE1-72
IE2-86
pp71
p21
CyclinD1
CDK4/6
+p
Rb
Cell cycle
p16INK4a
E2F
DNA
G1/S progression
TNF
TNFRI
TRADD
FADD
Casp8
FasL
Fas
UL36
Apoptosis
UL37x1
Mitochondrion
Bax
Bak
BID
Brockage of apoptotic pathway
CytC
CASP9
CASP3
05163 7/7/21
(c) Kanehisa Laboratories

H

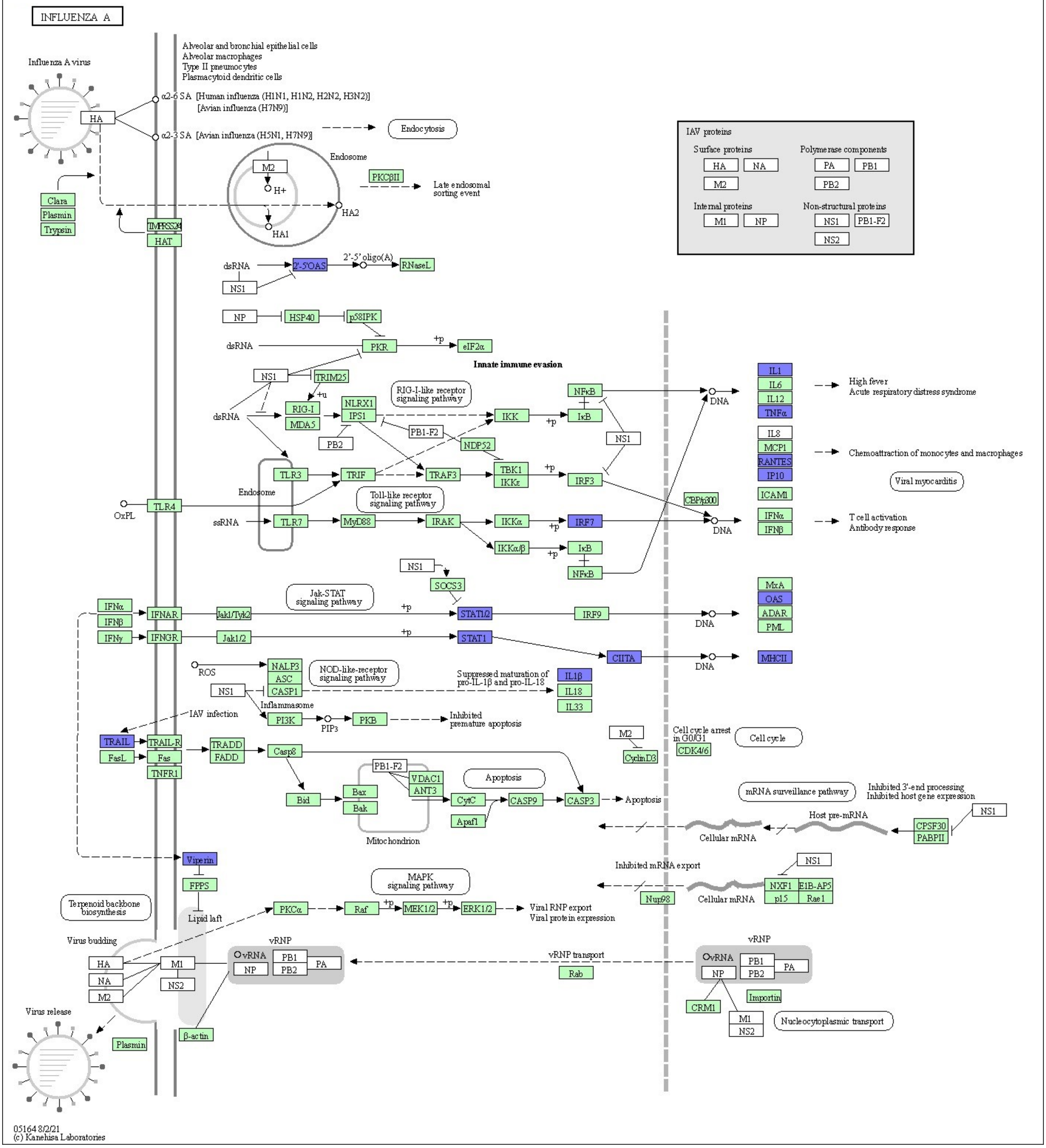

INFLUENZA A
Influenza A virus
Alveolar and bronchial epithelial cells
Alveolar macrophages
Type II pneumocytes
Plasmacytoid dendritic cells
HA
α2-6 SA [Human influenza (H1N1, H1N2, H2N2, H3N2)]
[Avian influenza (H7N9)]
α2-3 SA [Avian influenza (H5N1, H7N9)]
Endocytosis
Endosome
M2
H+
HA2
HA1
PKCβII
Late endosomal sorting event
Clara
Plasmin
Trypsin
TMPRSS2/4
HAT
IAV proteins
Surface proteins
HA
NA
M2
Polymerase components
PA
PB1
PB2
Internal proteins
M1
NP
Non-structural proteins
NS1
PB1-F2
NS2
dsRNA
2'-5'OAS
2'-5' oligo(A)
RNaseL
NS1
NP
HSP40
p58IPK
PKR
eIF2α
+p
Innate immune evasion
TRIM25
+u
RIG-I
MDA5
NLRX1
IPS1
RIG-I-like receptor signaling pathway
PB2
PB1-F2
NDP52
IKK
IκB
NFκB
TBK1
IKKε
IRF3
TLR3
TRIF
TRAF3
Endosome
TLR4
OxPL
ssRNA
TLR7
MyD88
IRAK
IKKα
IRF7
IKKα/β
Toll-like receptor signaling pathway
CBP/p300
DNA
IL1
IL6
IL12
TNFα
High fever
Acute respiratory distress syndrome
IL8
MCP1
RANTES
IP10
Chemoattraction of monocytes and macrophages
ICAM1
Viral myocarditis
IFNα
IFNβ
T cell activation
Antibody response
SOCS3
Jak-STAT signaling pathway
IFNα
IFNβ
IFNγ
IFNAR
IFNGR
Jak1/Tyk2
Jak1/2
STAT1/2
STAT1
IRF9
CIITA
MxA
OAS
ADAR
PML
MHCII
ROS
NALP3
ASC
CASP1
NOD-like-receptor signaling pathway
Inflammasome
Suppressed maturation of pro-IL-1β and pro-IL-18
IL1β
IL18
IL33
IAV infection
PI3K
PIP3
PKB
Inhibited premature apoptosis
TRAIL
TRAIL-R
FasL
Fas
TNFR1
TRADD
FADD
Casp8
Bid
Bax
Bak
VDAC1
ANT3
CytC
Apaf1
CASP9
CASP3
Apoptosis
Mitochondrion
Cell cycle arrest in G0/G1
CyclinD3
CDK4/6
Cell cycle
mRNA surveillance pathway
Inhibited 3'-end processing
Inhibited host gene expression
Host pre-mRNA
CPSF30
PABPII
Cellular mRNA
Inhibited mRNA export
NXF1
E1B-AP5
p15
Rae1
Nup98
Viperin
FPPS
Terpenoid backbone biosynthesis
Lipid laft
MAPK signaling pathway
PKCα
Raf
MEK1/2
ERK1/2
Viral RNP export
Viral protein expression
Virus budding
vRNP
vRNA
vRNP transport
Rab
CRM1
Importin
Nucleocytoplasmic transport
Virus release
β-actin
05164 8/2/21
(c) Kanehisa Laboratories

I

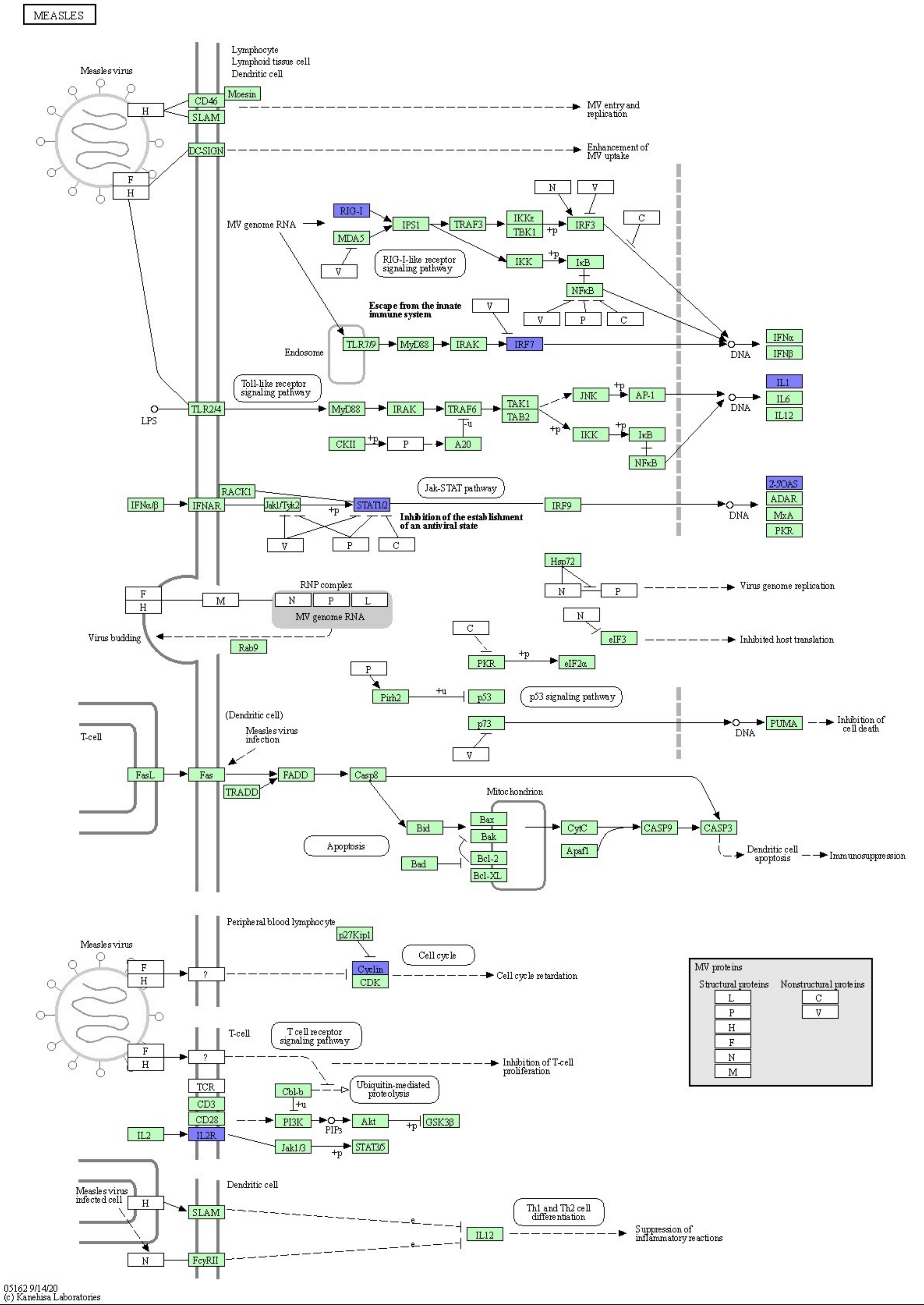
MEASLES
Measles virus
Lymphocyte
Lymphoid tissue cell
Dendritic cell
H
CD46
Moesin
SLAM
MV entry and replication
DC-SIGN
Enhancement of MV uptake
F
H
N
V
RIG-I
MV genome RNA
IPS1
TRAF3
IKKε
TBK1
IRF3
C
MDA5
V
RIG-I-like receptor signaling pathway
IKK
IκB
NFκB
Escape from the innate immune system
V
V
P
C
Endosome
TLR7/9
MyD88
IRAK
IRF7
DNA
IFNα
IFNβ
Toll-like receptor signaling pathway
LPS
TLR2/4
MyD88
IRAK
TRAF6
TAK1
TAB2
JNK
AP-1
DNA
IL1
IL6
IL12
CKII
P
A20
IKK
IκB
NFκB
RACK1
Jak-STAT pathway
IFNα/β
IFNAR
Jak1/Tyk2
STAT1/2
IRF9
DNA
2-5OAS
ADAR
MxA
PKR
Inhibition of the establishment of an antiviral state
V
P
C
Hsp72
N
P
Virus genome replication
RNP complex
F
H
M
N
P
L
MV genome RNA
N
eIF3
Inhibited host translation
Virus budding
Rab9
C
PKR
eIF2α
P
Pirh2
p53
p53 signaling pathway
p73
V
DNA
PUMA
Inhibition of cell death
(Dendritic cell)
T-cell
Measles virus infection
FasL
Fas
FADD
Casp8
TRADD
Mitochondrion
Bid
Bax
Bak
CytC
CASP9
CASP3
Apaf1
Apoptosis
Bad
Bcl-2
Bcl-XL
Dendritic cell apoptosis
Immunosuppression
Peripheral blood lymphocyte
p27Kip1
Measles virus
Cell cycle
F
H
?
Cyclin
CDK
Cell cycle retardation
MV proteins
Structural proteins
Nonstructural proteins
L
P
H
F
N
M
C
V
T-cell
T cell receptor signaling pathway
F
H
?
Inhibition of T-cell proliferation
TCR
CD3
CD28
Cbl-b
Ubiquitin-mediated proteolysis
PI3K
PIP3
Akt
GSK3β
IL2
IL2R
Jak1/3
STAT3/5
Measles virus infected cell
H
Dendritic cell
SLAM
Th1 and Th2 cell differentiation
IL12
Suppression of inflammatory reactions
N
FcγRII
05162 9/14/20
(c) Kanehisa Laboratories

J

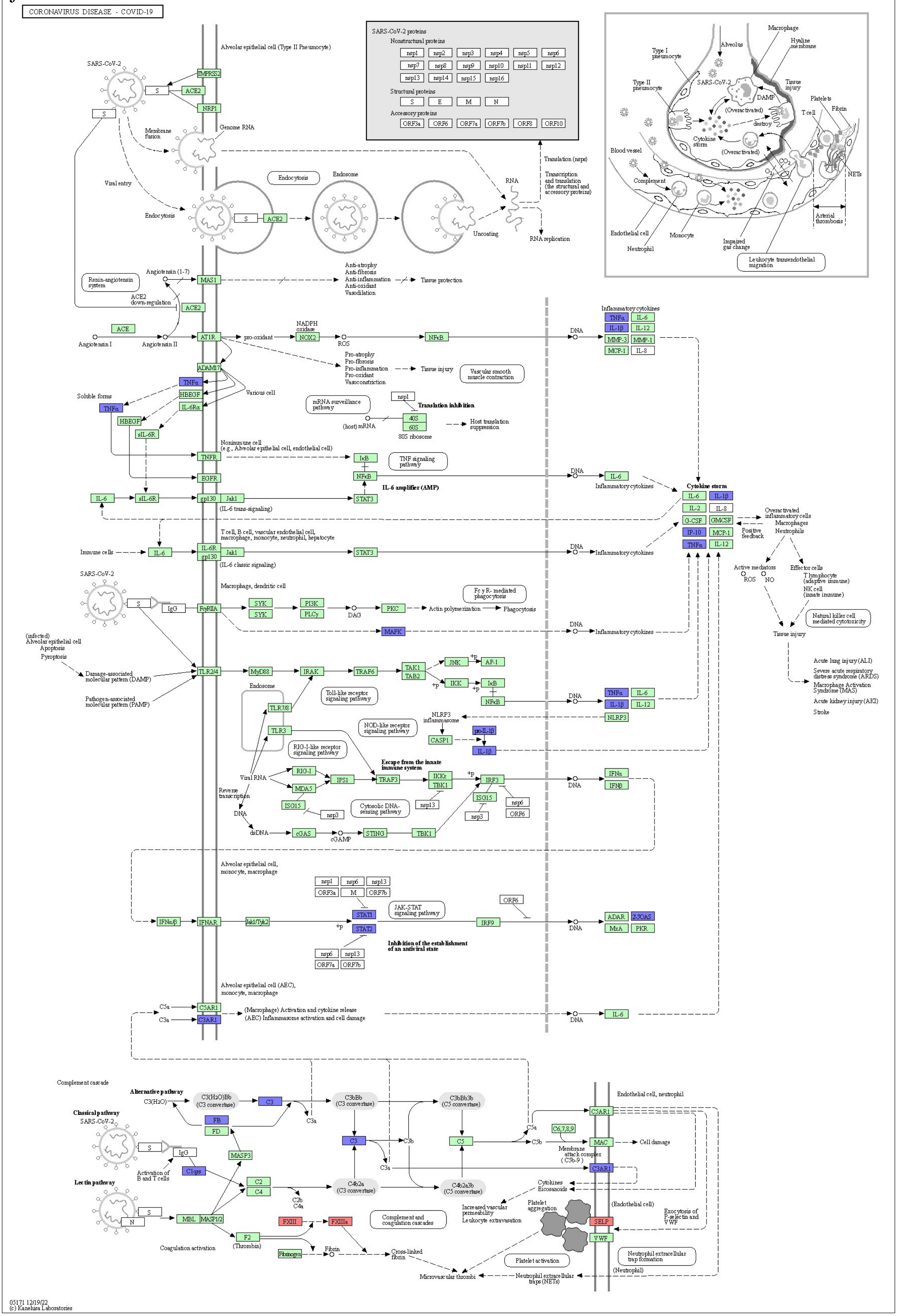

CORONAVIRUS DISEASE - COVID-19
SARS-CoV-2 proteins
Nonstructural proteins
nsp1 nsp2 nsp3 nsp4 nsp5 nsp6 nsp7 nsp8 nsp9 nsp10 nsp11 nsp12 nsp13 nsp14 nsp15 nsp16
Structural proteins
S E M N
Accessory proteins
ORF3a ORF6 ORF7a ORF7b ORF8 ORF10
Alveolar epithelial cell (Type II Pneumocyte)
TMPRSS2
ACE2
NRP1
Membrane fusion
Genome RNA
Viral entry
Endocytosis
Endosome
Uncoating
RNA
Translation (nsps)
Transcription and translation (the structural and accessory proteins)
RNA replication
Angiotensin (1-7)
MAS1
Renin-angiotensin system
ACE2 down-regulation
Anti-atrophy Anti-fibrosis Anti-inflammation Anti-oxidant Vasodilation
Tissue protection
Angiotensin I
Angiotensin II
ACE
AT1R
pro-oxidant
NADPH oxidase
NOX2
ROS
NFκB
Pro-atrophy Pro-fibrosis Pro-inflammation Pro-oxidant Vasoconstriction
Tissue injury
Vascular smooth muscle contraction
ADAM17
Various cell
Soluble forms
TNFα HBEGF IL-6Rα sIL-6R
mRNA surveillance pathway
Translation inhibition
(host) mRNA
40S 60S
80S ribosome
Host translation suppression
Nonimmune cell (e.g., Alveolar epithelial cell, endothelial cell)
TNFR
EGFR
IκB
TNF signaling pathway
IL-6 amplifier (AMP)
gp130 Jak1
STAT3
(IL-6 trans-signaling)
T cell, B cell, vascular endothelial cell, macrophage, monocyte, neutrophil, hepatocyte
Immune cells
IL-6R
(IL-6 classic signaling)
Inflammatory cytokines
TNFα IL-6 IL-1β IL-12 MMP-3 MMP-1 MCP-1 IL-8
DNA
Cytokine storm
IL-6 IL-1β IL-2 IL-8 G-CSF GMCSF IP-10 MCP-1 TNFα IL-12
Overactivated inflammatory cells
Macrophages
Neutrophils
Positive feedback
Active mediators
ROS NO
Effector cells
T lymphocyte (adaptive immune)
NK cell (innate immune)
Natural killer cell mediated cytotoxicity
Tissue injury
Acute lung injury (ALI)
Severe acute respiratory distress syndrome (ARDS)
Macrophage Activation Syndrome (MAS)
Acute kidney injury (AKI)
Stroke
Macrophage, dendritic cell
IgG
FcγRIIA
SYK PI3K PLCγ
DAG
PKC
Actin polymerization
Phagocytosis
FcγR-mediated phagocytosis
MAPK
(infected) Alveolar epithelial cell
Apoptosis
Pyroptosis
Damage-associated molecular pattern (DAMP)
Pathogen-associated molecular pattern (PAMP)
TLR2/4
MyD88
IRAK
TRAF6
TAK1 TAB2
JNK AP-1
IKK
Toll-like receptor signaling pathway
TLR7/8
TLR3
NLRP3 inflammasome
NOD-like receptor signaling pathway
CASP1
pro-IL-1β
NLRP3
RIG-I-like receptor signaling pathway
Escape from the innate immune system
Viral RNA
RIG-I MDA5
IPS1
TRAF3
IKKε TBK1
IRF3
IFNα IFNβ
ISG15
Reverse transcription
Cytosolic DNA-sensing pathway
dsDNA
cGAS
cGAMP
STING
Alveolar epithelial cell, monocyte, macrophage
IFNα/β
IFNAR
Jak1/Tyk2
STAT1 STAT2
JAK-STAT signaling pathway
IRF9
ADAR 2-5OAS MxA PKR
Inhibition of the establishment of an antiviral state
Alveolar epithelial cell (AEC), monocyte, macrophage
C5a C3a
C5AR1 C3AR1
(Macrophage) Activation and cytokine release
(AEC) Inflammasome activation and cell damage
Complement cascade
Alternative pathway
C3(H2O)
C3(H2O)Bb (C3 convertase)
FB FD
C3bBb (C3 convertase)
C3bBb3b (C5 convertase)
Classical pathway
C1qrs
MASP3
Activation of B and T cells
Lectin pathway
MBL MASP1/2
C2 C4
C4b2a (C3 convertase)
C4b2a3b (C5 convertase)
C6,7,8,9
MAC
Membrane attack complex (C5b-9)
Cell damage
Endothelial cell, neutrophil
Cytokines Eicosanoids
Increased vascular permeability
Leukocyte extravasation
Platelet aggregation
(Endothelial cell)
SELP
VWF
Exocytosis of P-selectin and VWF
FXIII FXIIIa
F2 (Thrombin)
Coagulation activation
Fibrinogen
Fibrin
Cross-linked fibrin
Complement and coagulation cascades
Platelet activation
Neutrophil extracellular trap formation
(Neutrophil)
Microvascular thrombi
Neutrophil extracellular traps (NETs)
Alveolus
Macrophage
Hyaline membrane
Type I pneumocyte
Type II pneumocyte
Tissue injury
DAMP
(Overactivated)
destroy
Platelets
Fibrin
T cell
Cytokine storm
Blood vessel
Complement
NETs
Arterial thrombosis
Endothelial cell
Neutrophil
Monocyte
Impaired gas change
Leukocyte transendothelial migration
05171 12/19/22
(c) Kanehisa Laboratories

Supplementary Figure S1. Dexamethasone-induced M2c macrophages exhibit immunosuppressive effects against parasites, bacteria, and viruses. The KEGG disease pathway maps of (A) Leishmaniasis, (B) toxoplasmosis, (C) tuberculosis, and (D) pertussis, (E) Herpes simplex virus 1 infection, (F) Kaposi sarcoma-associated herpesvirus infection, (G) human cytomegalovirus infection, (H) influenza A, (I) measles pathway, and (J) coronavirus disease. The upregulated and downregulated DEGs in the pathway are respectively denoted by red and blue colors.
